# SliC is a surface-displayed lipoprotein that is required for the anti-lysozyme strategy during *Neisseria gonorrhoeae* infection

**DOI:** 10.1371/journal.ppat.1007081

**Published:** 2018-07-05

**Authors:** Ryszard A. Zielke, Adriana Le Van, Benjamin I. Baarda, Marco F. Herrera, Christopher J. Acosta, Ann E. Jerse, Aleksandra E. Sikora

**Affiliations:** 1 Department of Pharmaceutical Sciences, College of Pharmacy, Oregon State University, Corvallis, Oregon, United States of America; 2 Department of Microbiology and Immunology, F. Edward Hebert School of Medicine, Uniformed Services University of the Health Sciences, Bethesda, Maryland, United States of America; University of Oxford, UNITED KINGDOM

## Abstract

Lysozymes are nearly omnipresent as the first line of immune defense against microbes in animals. They exert bactericidal action through antimicrobial peptide activity and peptidoglycan hydrolysis. Gram-negative bacteria developed several weapons to battle lysozymes, including inhibitors of c-type lysozymes in the MliC/PliC family and the *Neisseria* adhesin complex protein (ACP). Until the recent discovery of ACP, no proteinaceous lysozyme inhibitors were reported for the genus *Neisseria*, including the important human pathogen *N*. *gonorrhoeae*. Here, we describe a previously unrecognized gonococcal virulence mechanism involving a protein encoded by the open reading frame *ngo1063* that acts to counteract c-type Iysozyme and provides a competitive advantage in the murine model of gonorrhea. We named this protein SliC as a *s*urface-exposed *l*ysozyme *i*nhibitor of *c*-type lysozyme. SliC displays low overall primary sequence similarity to the MliC/PliC inhibitors, but we demonstrate that it has a parallel inhibitory mechanism. Our studies provide the first evidence that bacterial proteinaceous lysozyme inhibitors protect against host lysozyme during infection based on lack of attenuation of the Δ*sliC* mutant in lysozyme knock-out mice, and that the conserved residues involved in lysozyme inhibition, S83 and K103, are functionally indispensable during infection in wild type mice. Recombinant SliC completely abrogated the lytic activity of human and chicken c-type lysozymes, showing a preference towards human lysozyme with an IC_50_ of 1.85 μM and calculated K_D_ value of 9.2 ± 1.9 μM. In contrast, mutated SliC bearing S83A and K103A substitutions failed to protect fluorescein-labeled cell-wall from lysozyme-mediated hydrolysis. Further, we present data revealing that SliC is a surface-displayed lipoprotein released in membrane vesicles that is expressed throughout all phases of growth, in conditions relevant to different niches of the human host, and during experimental infection of the murine genital tract. SliC is also highly conserved and expressed by diverse gonococcal isolates as well as *N*. *meningitidis*, *N*. *lactamica*, and *N*. *weaveri*. This study is the first to highlight the importance of an anti-lysozyme strategy to escape the innate immune response during *N*. *gonorrhoeae* infection.

## Author summary

*Neisseria gonorrhoeae*, the etiologic agent of gonorrhea, is a clinically important pathogen due to the emergence of multi-drug resistance and the lack of a vaccine(s). During host colonization, pathogenic and commensal *Neisseria* inevitably encounter lysozyme, a major host innate defense factor that is abundantly present in epithelial secretions and phagocytic cells. Although *Neisseria* spp produce a c-type lysozyme inhibitor, the Adhesin Complex Protein, the significance of lysozyme inhibition for host colonization has not been addressed. Here we demonstrate the existence of a new c-type lysozyme inhibitor in *Neisseria*. We show that it is a surface-displayed lipoprotein in *N*. *gonorrhoeae* and, through its lysozyme-blocking function, plays a critical role in colonization of genital tract mucosae during infection in the female gonorrhea mouse model. We named the protein SliC as a *s*urface-exposed *l*ysozyme *i*nhibitor of *c*-type lysozyme. Understanding the mechanisms underlying anti-lysozyme strategies may facilitate antimicrobial development.

## Introduction

The first line of host immune defense against bacterial pathogens in plants and in invertebrate and vertebrate animals involves degradation of peptidoglycan through innate immune system components such as lysozymes [1–3]. Peptidoglycan (murein) is the major structural component of the bacterial cell envelope that provides resistance against turgor pressure and prevents cell death due to lysis. Peptidoglycan forms a giant three-dimensional network built of linear glycan strands of alternating β-(1,4) linked *N*-acetylmuramic acid and *N*-acetylglucosamine sugars that are cross-linked by short peptides [4]. Lysozymes are powerful host weapons exerting bacterial killing by hydrolytic action on the glycosidic bond linking the sugars, which breaks peptidoglycan [3, 5]. Three main classes of lysozymes have been distinguished within the animal kingdom including c- (conventional or chicken), g- (goose), and i- (invertebrate) type lysozyme [2, 3]. The mammalian lysozyme-like gene family consists of lysozyme c, lactalbumin and calcium-binding lysozyme [5]. Recent studies, however, showed the presence of eight additional diverse types of lysozyme-like genes in the genome of the common ancestor of all extant mammals, and ten lysozyme-like sequences distributed over five chromosomes in humans [6]. Therefore, it is not surprising that bacteria have evolved sophisticated mechanisms to escape killing via murein hydrolysis by chemical modifications of the peptidoglycan backbone and by synthesis of proteinaceous lysozyme inhibitors [7, 8]. The latter anti-lysozyme strategy targets i-, g-, or c-type lysozymes and encompasses at least five inhibitor families distributed exclusively in Gram-negative bacteria and predominantly in Proteobacteria [8]. Among the inhibitors of c-type lysozyme are Ivy (*I*nhibitor of *v*ertebrate *ly*sozyme) and Ivy-like proteins, the MliC/PliC family (*m*embrane-bound or *p*eriplasmic *l*ysozyme *i*nhibitor of *c*-type lysozyme, respectively), and the *Neisseria* *A*dhesin *C*omplex *P*rotein, ACP [8, 9].

MliC/PliC proteins and ACP have been implicated in host colonization based on their ability to at least partially protect bacteria against challenge with their cognate lysozyme *in vitro* [8–12]. Surprisingly, to date, only two reports have investigated the role of lysozyme inhibitors in *in vivo* virulence. These studies demonstrated the importance of MliC from Avian Pathogenic *Escherichia coli* and *Edwardsiella tarda* in a subcutaneous chicken infection model and a turbot model, respectively [13, 14].

*N*. *gonorrhoeae* colonizes mucosal surfaces of the reproductive tract, pharynx, rectum and conjunctiva in its sole human host (15). During colonization of these different niches, the gonococcus may encounter c-type lysozyme in tears, saliva, urine, serum, vaginal fluid, cervical mucus, and in the lysosomal granules of neutrophils and macrophages (3, 16, 17). Until the recent discovery of ACP, no proteinaceous lysozyme inhibitors were reported for the genus *Neisseria*, including the important human pathogen, *N*. *gonorrhoeae* [8, 9].

Here we describe a previously unrecognized *N*. *gonorrhoeae* virulence mechanism involving a protein encoded by the open reading frame (ORF) *ngo1063* in *N*. *gonorrhoeae* FA1090 that acts as a weapon to counteract the innate immunity effector c-type lysozyme and significantly impacts *N*. *gonorrhoeae* fitness in the female mouse genital tract. We identified NGO1063 in high-throughput proteomic investigations geared towards the discovery of potential gonorrhea vaccine antigens and therapeutic targets in *N*. *gonorrhoeae* cell envelopes and naturally released membrane vesicles (MVs) [15, 16]. NGO1063 is annotated in UniProt as a membrane-bound lysozyme inhibitor of the c-type lysozyme family (Protein Q5F7V4_NEIG1). NGO1063 displays low overall sequence similarity to the MliC/PliC protein family, but we demonstrate that it has a parallel inhibitory mechanism and provide the first evidence that the conserved residues involved in lysozyme inhibition are functionally critical during *in vivo* infection. Supporting these findings, gonococci lacking NGO1063 show comparable fitness to wild type bacteria during competitive infection in lysozyme-defective mice, but are attenuated in wild-type mice of the same background. We also present data showing that NGO1063 is exposed to the extracellular milieu, expressed during *in vivo* infection, and conserved in pathogenic and commensal *Neisseria*, highlighting the importance of this anti-lysozyme strategy during host colonization. We named this protein SliC as a *s*urface-exposed *l*ysozyme *i*nhibitor of *c*-type lysozyme.

## Results

### NGO1063 is a putative lysozyme c inhibitor

**Fig 1:**
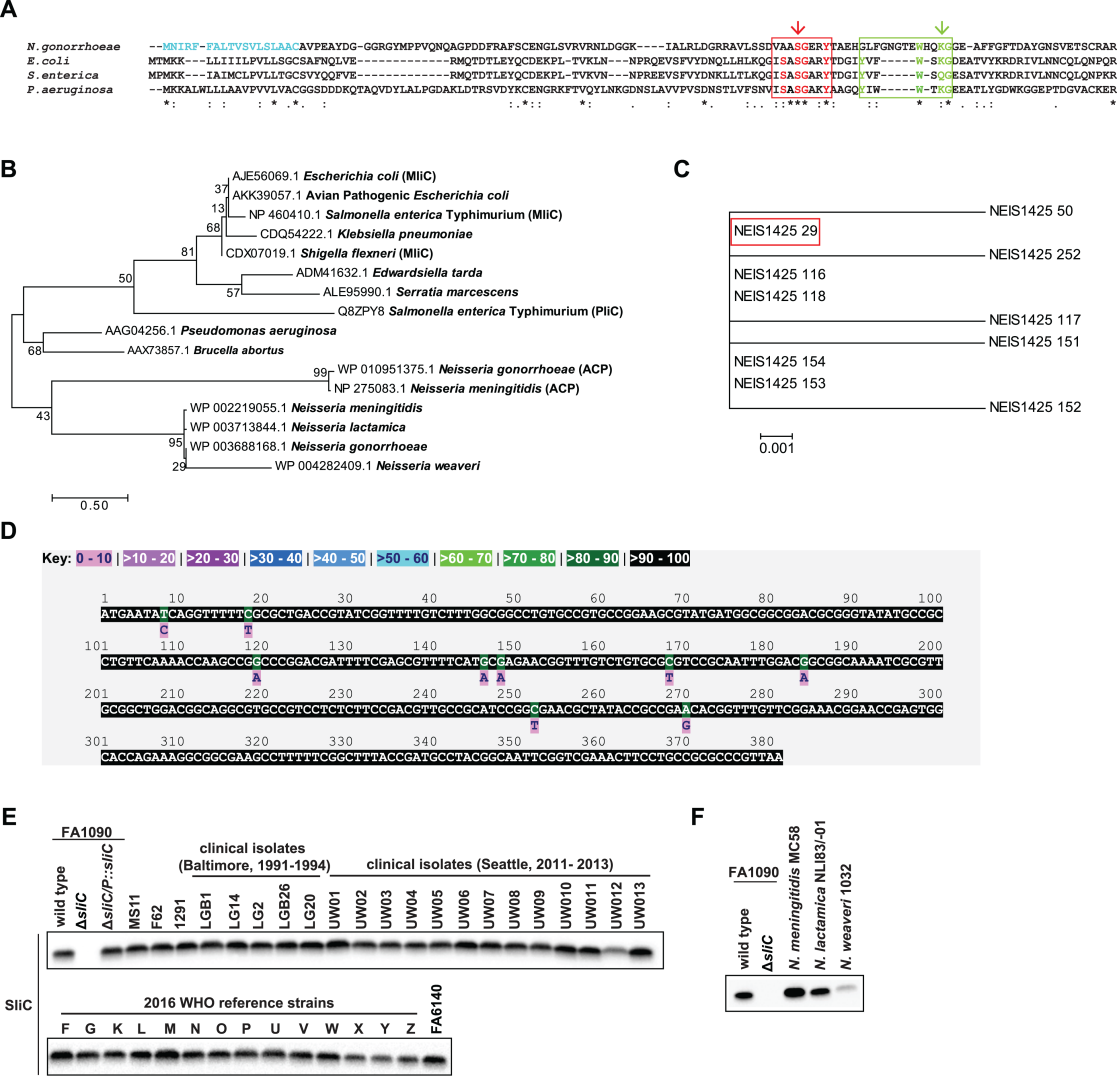
Sequence alignment, phylogenetic analysis and conservation of SliC. (A) The amino acid sequence of SliC from *N*. *gonorrhoeae* FA1090 (WP 003688168) was aligned with MliC for *E*. *coli* (AJE56069), *S*. *enterica* (NP 460410), and *P*. *aeruginosa* (AAG04256) sequences downloaded from NCBI with the Clustal Omega online tool (Clustal 2.1; https://www.ebi.ac.uk/Tools/msa/clustalo/) using the default alignment parameters. The predicted lipoprotein signal peptide is shown in aquamarine. The conserved regions of the COG3895 domain in the MliC/PliC family, SxSGAxY and YxxxTKG are shown in red and green boxes, respectively. Preserved residues are marked in color within conserved regions and by asterisks (*) throughout the sequences. The residues S83 and K103, predicted to be involved in interaction with c-type lysozyme are designated by red and green arrows, respectively. (B) A phylogenetic comparison of SliC, MliC/PliC and ACP. The tree was constructed using the Jones-Taylor-Thornton model [58] in MEGA. Neighbor-Join and BioNJ algorithms were applied to a pairwise-distance matrix derived from the JTT model to obtain an initial tree for the heuristic search. The phylogenies were tested by 500 bootstrap iterations, and the tree with the highest log likelihood is presented. (C) A phylogenetic comparison of SliC alleles in 3787 isolates of *N*. *gonorrhoeae* deposited to the PubMLST was performed as described above. The dominant allele (29) present in over 97% gonococcal isolates and in FA1090 is denoted in a red box. (D) Analysis of single nucleotide polymorphisms of SliC (locus NEIS1425) in *N*. *gonorrhoeae* was performed by comparing DNA sequences between gonococcal isolates deposited to PubMLST (https://pubmlst.org/neisseria/, as of April 11, 2018). (E) A panel constituting 37 *N*. *gonorrhoeae* isolates that are distinct genetically, geographically, and temporally, including common laboratory strains; clinical isolates collected from public health clinics in Baltimore from 1991 to 1994 and Seattle from 2011 to 2016; and the 14 isolates constituting the 2016 WHO reference strains were grown concurrently on solid medium for 20 h in 5% CO_2_ at 37° C. Cells were collected, lysed and processed for immunoblotting. (F) Whole-cell lysates of different *Neisseria*, including pathogenic *N*. *meningitidis* MC58, commensal *N*. *lactamica* NLI83/-01, and a human opportunistic pathogen *N*. *weaveri* 1032 were separated by SDS-PAGE and probed with antisera. In all experiments, samples were matched by equivalent OD_600_ units, resolved in a 4 to 20% Tris-glycine gel, and transferred onto nitrocellulose. Immunoblots were performed using polyclonal rabbit antisera against SliC. Cell lysates of wild type FA1090 and isogenic **Δ***sliC* were loaded as controls for the experiments.

**Fig 2:**
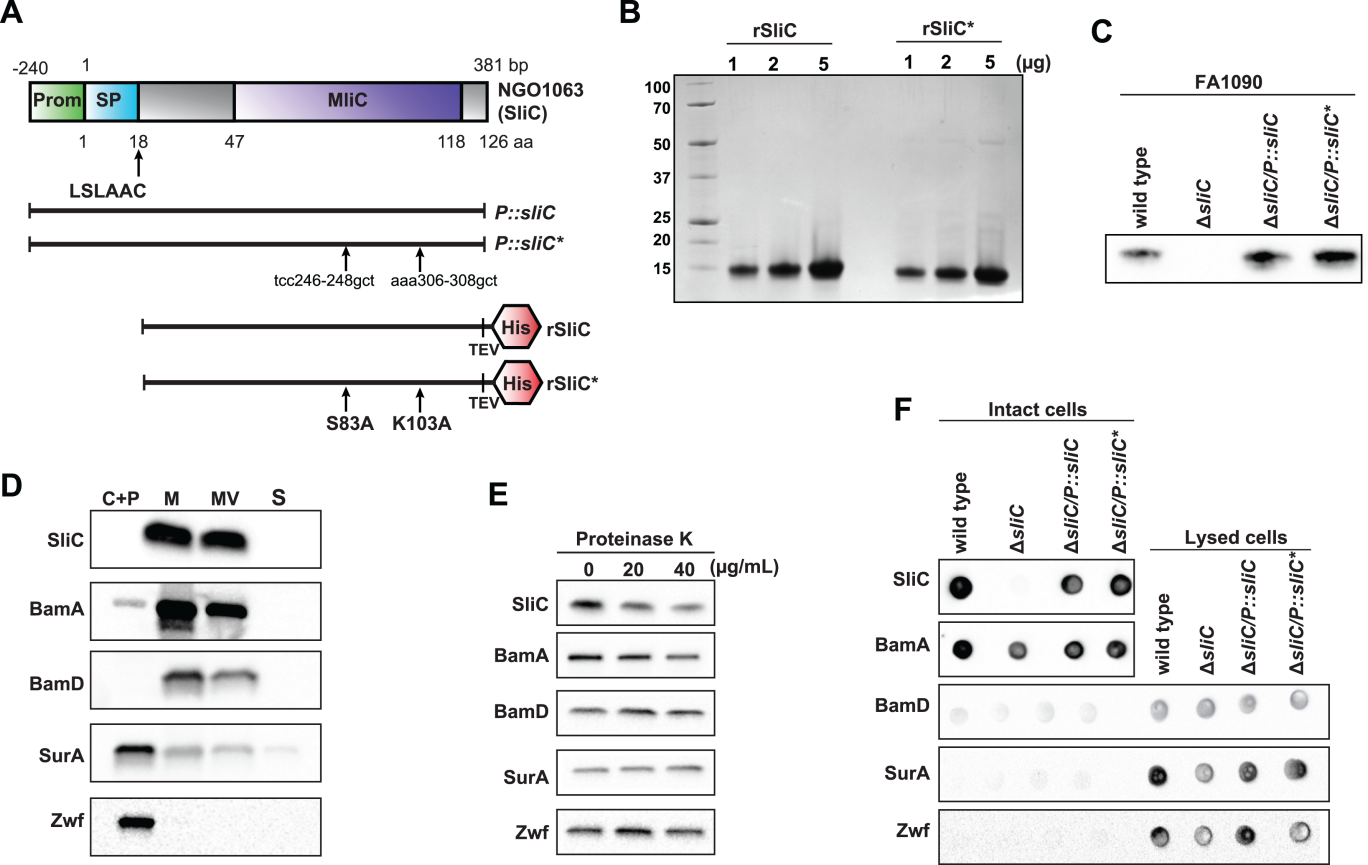
SliC is a surface-displayed lipoprotein released in natural membrane vesicles. (A) Diagrams of native SliC, constructs for complementation studies using wild type and mutated versions of *sliC* placed under the control of the native promoter (*P*::*sliC* and *P*::sliC*, respectively), and recombinant versions of wild type SliC (rSliC), and its mutated variant (rSliC*) bearing alanine substitutions in residues S83 and K103 are shown. Introduced mutations are denoted by arrows. Both recombinant proteins lack a signal peptide and contain a C-terminal Tobacco Etch Virus (TEV) protease cleavage site followed by a C-terminal 6×His-tag (His). The *sliC* native promoter (Prom) is shown in green; the lipoprotein signal peptide (SP) in aquamarine; the MliC/PliC domain in lavender. Amino acid (aa) domain positions are denoted. (B) Recombinant versions of matured rSliC and rSliC* were purified in a soluble form to homogeneity through affinity and size exclusion chromatography steps and untagged proteins were separated on SDS-PAGE. Proteins were stained with colloidal coomassie. Samples were loaded by protein concentration, as indicated above the gel. Migration of a molecular mass marker (kDa) is indicated on the left. (C) Strains of *N*. *gonorrhoeae* FA1090 wild type, isogenic null mutant in *ngo1063* (**Δ***sliC*), and complemented strains, **Δ***sliC*/*P*::*sliC* and **Δ***sliC*/*P*::sliC*, harboring either wild type *sliC* or mutated sliC* bearing S83A K103A substitutions, respectively, were collected from solid media, lysed, separated by SDS-PAGE, and probed with polyclonal rabbit anti-SliC antisera. Whole cell lysates were matched by the same OD_600_. (D) Cultures of *N*. *gonorrhoeae* FA1090 wild type harvested from liquid cultures at mid-logarithmic growth were subjected to subcellular fractionation. Isolated proteomes of cytoplasmic and periplasmic (C+P), cell envelopes (M), naturally released membrane vesicles (MV), and soluble supernatant fractions (S) were matched by the same total protein concentration, separated by SDS-PAGE and probed with polyclonal rabbit antisera against SliC, BamA, BamD, SurA, and Zwf. (E) Wild type gonococci were collected from solid media, sub-cultured in broth for 3 h, diluted to OD_600_ of 0.1, and cultured until OD_600_ of ~ 1.0 was reached. Gonococci were washed and after OD_600_ adjustment to 2.0, the bacterial cells were incubated for 1 h at 37°C with proteinase K at final concentrations as designated above the immunoblots. Protease was deactivated, samples were normalized by the same OD_600_, subjected to SDS-PAGE, and probed with polyclonal antisera to determine protease susceptibility. (F) *N*. *gonorrhoeae* FA1090 strains, as shown above the graphs, were cultured in liquid medium, harvested, and suspended to OD_600_ of 2.0. Intact and lysed cells were spotted onto nitrocellulose membranes. Individual protein profiles were analyzed by immunoblotting with specific antisera against SliC, BamA, BamD, SurA, and Zwf.

Members of different lysozyme inhibitor families show little similarity at the primary amino acid sequence level [8]. Despite the low sequence identity (24–39%) of the PliC and MliC type inhibitors, these inhibitors display the same specificity and share a specific sequence motif as well as an eight-stranded antiparallel ß-barrel structural topology [8, 11, 12]. *In silico* analysis of the deduced amino acid sequence of our new proteome-derived antigen/drug target [15, 16], *sliC*, revealed a predicted signal peptide with a lipobox motif (LSLAAC) containing an invariant cysteine (C18) and a putative MliC domain (residues 47–118; Figs 1A and 2A). Comparison of the primary amino acid sequence of SliC with other characterized MliC proteins from *E*. *coli*, *Salmonella enterica* and *Pseudomonas aeruginosa* showed only 18.8–23.3% overall identity (S1 Table). However, the additional conserved regions of the COG3895 domain in the MliC/PliC family, SxSGAxY and YxxxTKG [8], closely resembled those found in SliC (Fig 1A). Further, the *P*. *aeruginosa* MliC residues S89 and K103, involved in interaction with hen egg white c-type lysozyme (HEWL) [11], were present in SliC (S83 and K103), which together suggested that *ngo1063* encodes a c-type lysozyme inhibitor.

To further compare SliC to other lysozyme inhibitors of the MliC/PliC family and *Neisseria* ACP, a phylogenetic analysis was performed (Fig 1B). Two distinct clusters were revealed in the phylogenetic tree; one cluster contained lysozyme inhibitors found in the genomes of *Neisseria* species, while the second cluster was comprised of MliC and PliC proteins from more diverse bacterial species. These results indicated that meningococcal and gonococcal SliC and ACP are more closely related to each other than to MliC/PliC lysozyme inhibitors from other bacterial species, despite their low primary sequence identity (S1 Table).

### SliC is conserved and expressed in a panel of diverse *Neisseria*

To extend these studies, we analyzed SliC sequence diversity among *N*. *gonorrhoeae* and other *Neisseria* species using the translated amino acid sequences from 10 alleles found in *N*. *gonorrhoeae* (Fig 1C) and the 224 alleles found in the population of over 44 thousand *Neisseria* isolates present in the PubMLST database (S1 Fig). These phylogenetic analyses showed that all SliC alleles are closely related in pathogenic and commensal *Neisseria* and allele 29, of which FA1090 SliC is a member (Fig 1C, red box), represented nearly 98% of the alleles found in *N*. *gonorrhoeae* (3,787 out of 4,990 *N*. *gonorrhoeae* isolates; 1,111 isolates had no value for the *sliC* locus). Analysis of single nucleotide polymorphisms revealed the existence of 9 and 157 polymorphic sites within all alleles found in *N*. *gonorrhoeae* (Fig 1D) and in all *Neisseria* (S2 Fig), respectively, further confirming high conservation of SliC.

Finally, to assess the conservation at the level of immune-recognition, we probed whole cell lysates from 37 temporally, geographically and genetically diverse *N*. *gonorrhoeae* isolates, including common laboratory strains; clinical isolates collected from public health clinics in Baltimore from 1991 to 1994 and Seattle from 2011 to 2013 [16]; and the 2016 WHO reference strains [17] with antiserum against FA1090 SliC (Fig 1E). SliC was detected in all strains tested, and in pathogenic *N*. *meningitidis*; commensal *N*. *lactamica*; and a human opportunistic pathogen typically associated with canine bite wounds, *N*. *weaveri* (Fig 1F).

Cumulatively, these findings demonstrated a high conservation of SliC and a similar cellular pool in pathogenic *N*. *gonorrhoeae and N*. *meningitidis*.

### Purification of SliC variants and mutant construction

Lysozyme inhibitors in their natural forms are difficult to obtain with high purity, therefore these proteins have been examined primarily as recombinant proteins [9, 12, 14, 18, 19]. To initiate characterization of SliC, we engineered recombinant versions of wild type SliC (rSliC) and its mutated variant (rSliC*) bearing alanine substitutions in residues S83 and K103. Both recombinant proteins lacked signal peptides and contained a C-terminal Tobacco Etch Virus (TEV) protease cleavage site followed by a 6×His-tag (Fig 2A). The two proteins were purified in a soluble form to homogeneity through several chromatography steps and migrated on SDS-PAGE according to the predicted molecular mass of 11.65 kDa (Fig 2B). Untagged rSliC was used to obtain polyclonal rabbit antisera. Subsequently, we utilized *N*. *gonorrhoeae* FA1090 to create a null mutant in *ngo1063* (**Δ***sliC*) and complemented strains, **Δ***sliC*/*P*::*sliC* and **Δ***sliC*/*P*::sliC*, in which either wild type *sliC* or mutated sliC* bearing S83A K103A substitutions were expressed under the control of the native *sliC* promoter (Fig 2A). The polyclonal rabbit anti-SliC antisera recognized SliC within whole cell lysates obtained from wild type and both complemented strains, but no signal was detected in **Δ***sliC* (Fig 2C).

### SliC is a surface-displayed lipoprotein released in natural membrane vesicles

Lipoproteins traverse the cell envelope and may be anchored through their invariant triacylated cysteine residue to the periplasmic side of the inner or outer membrane, or may be translocated across the outer membrane for cell-surface decoration [20–22]. To examine SliC localization, cell sub-fractionation experiments coupled with immunoblotting were performed. Corroborating our proteomic investigations [15, 16], these experiments demonstrated the presence of SliC in the cell envelopes and naturally released membrane vesicles. The outer membrane proteins BamA and BamD were also present in this fraction, and as expected, the periplasmic and cytoplasmic markers, SurA and Zwf, respectively, were found primarily in the periplasmic and cytoplasmic sub-proteomes (Fig 2D).

Previously applied *in silico* approaches categorized SliC as an extracellular protein [15]; however, a reliable method to predict whether a given lipoprotein is surface-exposed or faces the periplasmic side of the outer membrane is currently lacking [22]. Therefore, we utilized proteinase K shaving assays and dot blots as two independent experimental methodologies to further examine SliC localization. Exposing intact gonococci to increasing concentrations of proteinase K resulted in detection of lower amounts of SliC, indicating that it is accessible to external protease, similarly to the surface protein marker BamA (Fig 2E). In contrast, BamD, which is anchored to the periplasmic side of the outer membrane [23], remained unaffected. Likewise, SurA and Zwf resisted proteinase K challenge, verifying the intactness of *N*. *gonorrhoeae* cells during the experimental procedures. SliC was also detected on the cell surface of wild type, **Δ***sliC*/*P*::*sliC*, and **Δ***sliC*/*P*::sliC* when analyzed by an immunological dot blot assay, in which suspensions of intact gonococci are spotted on nitrocellulose filters followed by incubation with the antisera (Fig 2F). In these experiments, as expected, BamA was detected on the cell surface in all strains, whereas no signal was observed for BamD, SurA and Zwf unless the cells were lysed.

Together, these studies showed that SliC is a surface-displayed lipoprotein released to the extracellular milieu in native membrane vesicles and that alteration of residues predicted to interact with c-type lysozyme does not affect its localization.

### SliC functions as a lysozyme inhibitor

**Fig 3:**
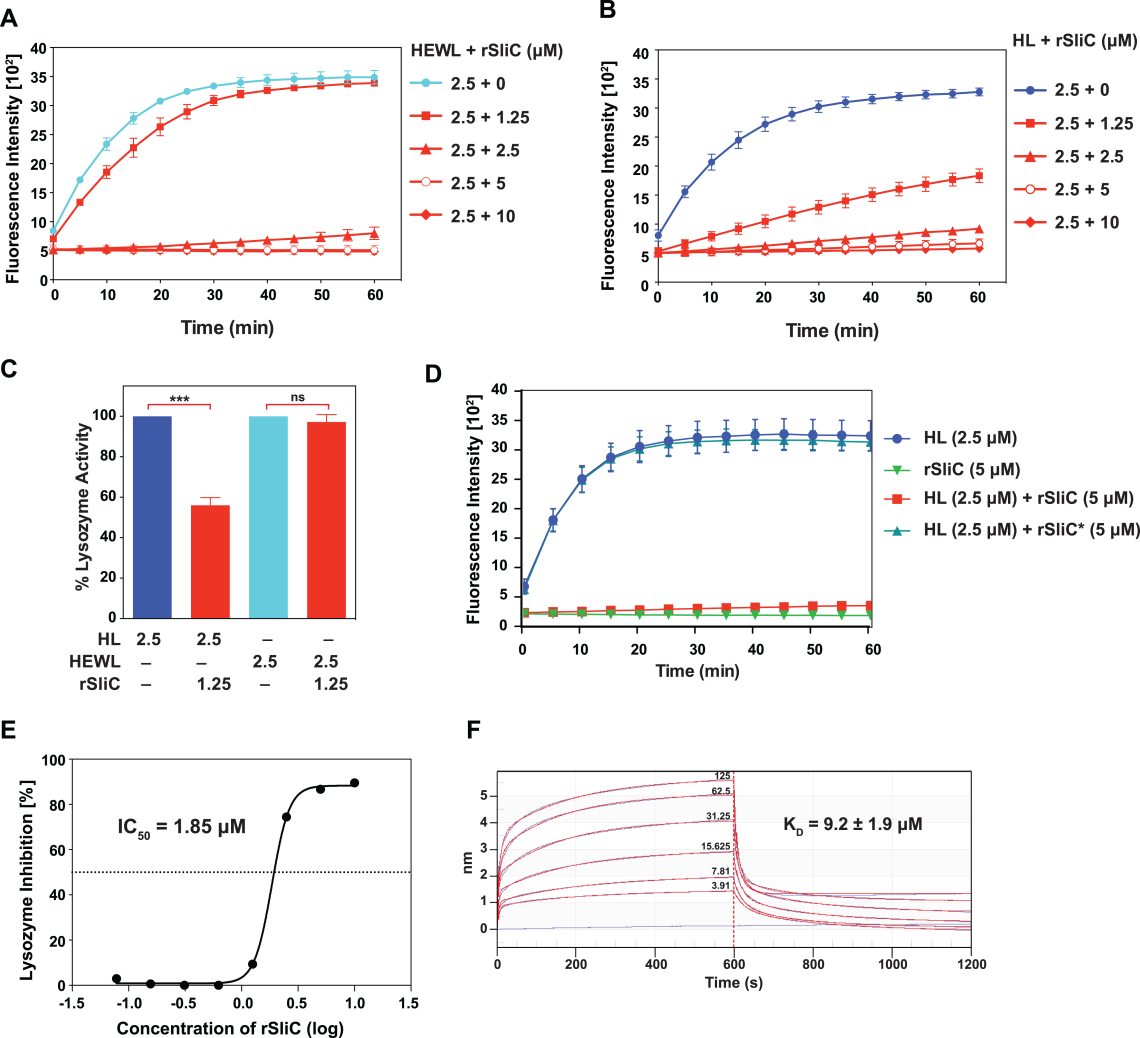
Biochemical characterization of SliC-c type lysozyme interaction. (A-E) Lysozyme activity assays with fluorescein-labeled cell walls of *Micrococcus lysodeikticus* as lysozyme substrate were used to determine the SliC-mediated inhibition of hen egg white c-type lysozyme (HEWL; A, C) and human lysozyme (HL; B-E). All assays were conducted using 96 well microtiter plates. Experimental samples containing 2.5 μM of either HEWL or HL were incubated with increasing concentrations of SliC (0–10 μM; as indicated on the right of each panel). The control wells contained HEWL, HL, or SliC alone. After incubation, the reaction was initiated by addition of lysozyme substrate and monitored for 1 h at excitation and emission wavelengths of 485 nm and 530 nm, respectively. Experiments reported in panels A and B show averages and corresponding SEMs from four independent trials. (C) Comparison of the inhibition of HEWL and HL activity by incubation with molar equivalent concentrations of rSliC (*n* = 4, mean±SEMs) was performed as described above. Protein concentrations (μM) are shown below the graph. ***p<0.0001; not statistically significant *p* value of 0.25 (ns). (D) 2.5 μM of HL and 5 μM of either SliC or SliC* were used to test the inhibition of HL by SliC*. Assays were performed as outlined above (*n* = 3, mean±SEMs). (E) The half-maximal (50%) inhibitory concentration, IC_50_, of SliC against the lytic activity of HL towards *M*. *lysodeikticus* peptidoglycan was determined by incubating 2.5 μM of HL with increasing concentrations of SliC (0–10 μM). (F) The binding affinity of SliC to lysozyme was assessed by biolayer interferometry. Biotinylated rSliC (20 μg/mL) was immobilized on streptavidin sensors for 10 min and incubated with increasing concentrations of HL as shown on the graph. Unloaded sensors were used as controls. After establishing the baseline, the association and dissociation steps were performed for 600 s. Experiments were performed in three biological replicates with curve fitting using a 2:1 (Heterogeneous Ligand) model. K_D_ value calculations were completed using Octet Data Analysis and mean±SEM is reported (version 9).

To examine whether SliC acts as an inhibitor of c-type lysozyme, we employed an assay that takes advantage of a highly sensitive quenched fluorescent lysozyme substrate–fluorescein-labeled cell walls of *Micrococcus lysodeikticus*. As expected, addition of either HEWL or human lysozyme c (HL) to the bacterial suspension triggered hydrolysis of the ß-(1,4) bond between *N*-acetylmuramic acid and *N*-acetylglucosamine in peptidoglycan and resulted in robust fluorescence dequenching (Fig 3A, 3B and 3D). These reactions were abrogated upon addition of increasing amounts of rSliC. Comparison of the inhibition of HEWL and HL activity by incubation with molar equivalent concentrations of rSliC demonstrated drastically reduced HL lytic activity in the presence of 1.25 μM rSliC, whereas no significant effect was observed for HEWL (Fig 3C). Furthermore, the mutated SliC variant, rSliC*, failed to inhibit HL-driven cell-wall hydrolysis, confirming that S83 and K103 were critical residues for the SliC-HL interaction (Fig 3D). The half-maximal (50%) inhibitory concentration, IC_50_, of SliC against the lytic activity of HL towards *M*. *lysodeikticus* peptidoglycan was determined to be 1.85 μM (Fig 3E).

To gain further insights into interactions between SliC and HL, we employed Bio-Layer Interferometry (BLI), a label-free biophysical method that provides kinetic data for protein-protein interactions. It is similar to Surface Plasmon Resonance but is less affected by changes in sample composition [24, 25]. Biotinylated rSliC was immobilized on streptavidin sensors and incubated with increasing concentrations of HL. BLI experiments were executed using a steady state method and curve fitting of the association and dissociation responses. Curves were fitted to a biphasic binding model, yielding a calculated K_D_ value of 9.2 ± 1.9 μM (average ± SEM; Fig 3F). This result suggests a moderate binding of SliC to HL *in vitro*, which is very similar to the ACP-HL interaction with a K_D_ of 11 μM [9].

Cumulatively, our biochemical studies demonstrated that SliC is an efficient inhibitor of c-type lysozyme with functionally pivotal residues S83 and K103 and suggest that SliC is a better inhibitor of HL than HEWL.

### SliC does not affect *N*. *gonorrhoeae* fitness *in vitro*

**Fig 4:**
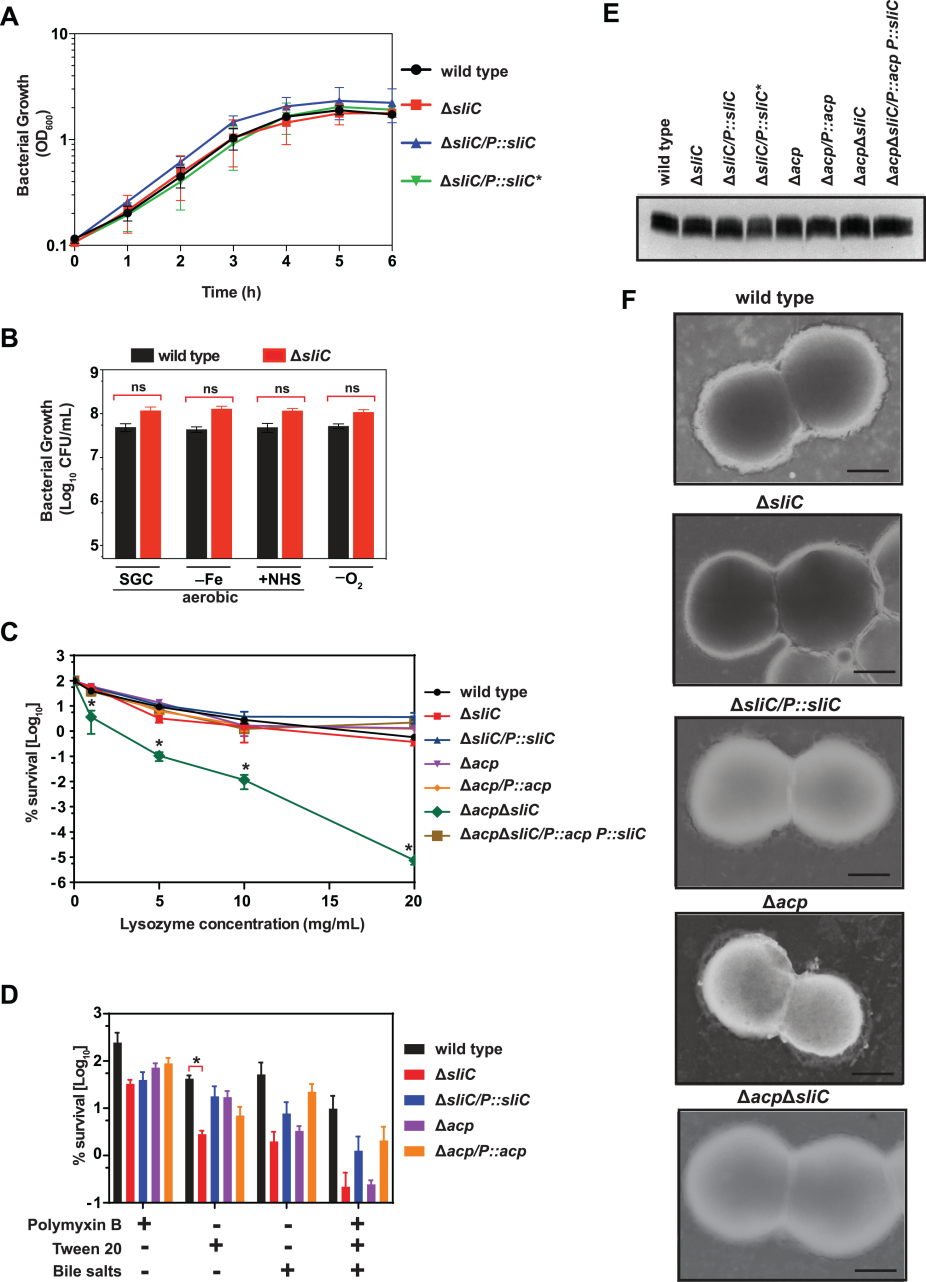
Fitness assessment of *N*. *gonorrhoeae* lacking functional SliC and ACP. (A) *N*. *gonorrhoeae* FA1090 wild type, isogenic Δ*sliC*, Δ*sliC*/*P*::*sliC*, and Δ*sliC*/*P*::sliC* were harvested from solid media, suspended to OD_600_ = 0.1 and incubated under standard aerobic conditions for 3 h. Then, cultures were back-diluted in fresh media and their growth was monitored by measuring OD_600_ at indicated time points for 6 h. (B) Bacterial strains were collected from solid media, suspended to an OD_600_ of 0.1, serially diluted and plated for fitness assessments under standard growth conditions on solid media (SGC), during iron limitation (‐Fe), in the presence of 7.5% normal human serum (+NHS), and anaerobically (‐O_2_). CFU’s were enumerated after 22 h of aerobic and 48 h of anaerobic growth. Experiments were performed in three biological replicates and means with corresponding SEMs are presented. (C) To assess the role of SliC and ACP in *in vitro* protection of *N*. *gonorrhoeae* from the hydrolytic activity of HL, wild type FA1090, Δ*sliC*, Δ*sliC* complemented with *sliC*, Δ*acp*, Δ*acp/P*::*acp*, Δ*acpΔsliC*, or Δ*acpΔsliC/P*::*acp P*::*sliC* were suspended in liquid media to an OD_600_ of 0.1 and cultured at 37°C for 3 h. Bacteria were diluted to 1×10^4^ CFU/mL and 90 μL of each culture was incubated for 3 h at 37°C with the membrane-permeabilizing agent lactoferrin (5 mg/mL) and increasing concentrations of human lysozyme as specified below the graph. Suspensions were plated for CFU scoring (*n* = 5, mean±SEMs; *p<0.05). (D) Gonococci lacking SliC or ACP were challenged with cell envelope membrane permeabilizing agents (Polymyxin B at 100 U/mL; Tween 20 at 0.001%; bile salts at 0.05%; or all compounds combined together) in the presence of lactoferrin (5 mg/mL) and lysozyme (5 mg/mL) as described above and bacteria were plated for CFU scoring after 4 h of incubation at 37°C (*n* = 3, mean±SEMs; *p<0.05). (E) Lipooligosaccharide was isolated from indicated *N*. *gonorrhoeae* strains collected from solid media. After cell lysis and proteinase K treatment equal amounts were loaded on SDS-PAGE followed by silver staining. (F) Scanning electromicrographs of *N*. *gonorrhoeae* wild type FA1090, Δ*sliC*, Δ*sliC* complemented with *sliC*, Δ*acp*, and Δ*acpΔsliC*. Gonococci were prepared from exponentially-growing cultures, washed in PBS, spotted on copper grids and negatively stained with phosphotungstic acid. Scale bar represents 0.5 μm.

Complete elimination of SliC or replacing SliC with its mutated variant, SliC*, expressed at the native level (Fig 2C) had no effect on the growth rate of **Δ***sliC* or **Δ***sliC/P*::sliC* strains in liquid media (Fig 4A). Similarly, no fitness defects were observed when Δ*sliC* was exposed to conditions that more closely resemble micro-ecological niches encountered by gonococci in the human host such as iron deprivation, the presence of normal human sera and anaerobiosis (Fig 4B).

We next studied whether SliC protects *N*. *gonorrhoeae* from the hydrolytic activity of HL in the presence of the outer membrane permeabilizing protein, lactoferrin. Surprisingly, even at HL concentrations higher than are physiologically relevant, absence of SliC did not render the cells more susceptible (Fig 4C). We hypothesized that the lysozyme-blocking function of ACP may contribute to the observed phenomenon [9, 26, 27]. To dissect the possible functional relationship between SliC and ACP, we constructed an *acp* clean knockout and **Δ***acp***Δ***sliC* mutant in the parental FA1090 background. Subsequently, all mutant strains concurrently with the corresponding complemented strains were incubated with lactoferrin at 5 mg/mL and increasing concentrations of lysozyme. The single **Δ***sliC* and **Δ***acp* mutants had similar sensitivity towards lysozyme as the wild type cells while the double mutant exhibited 70, 370 and 67,000 fold reduction in survival when exposed to 5, 10 and 20 mg/mL of lysozyme, respectively (Fig 4C; green line). This sensitivity phenotype was restored to wild type resistance in the complemented strain carrying *acp* and *sliC* placed under the control of their own promoters. These studies suggested that SliC and ACP work interchangeably and each of the lysozyme inhibitors, in addition to the other mechanisms used by *N*. *gonorrhoeae* to resist killing by lysozyme e.g. lytic transglycosylases [9, 26, 27], is sufficient to provide resistance to lysozyme *in vitro*. Exposure of **Δ***sliC* and **Δ***acp* knockouts to additional cell envelope permeabilizing agents (antimicrobial peptide polymyxin B, bile salts, or nonionic surfactant Tween 20) in combination with lysozyme and lactoferrin treatment resulted in statistically significant decrease in viability of **Δ***sliC* bacteria solely in the presence of the non-physiologically-relevant Tween 20 (Fig 4D).

To test whether lack of the surface–exposed SliC, ACP or both proteins have effects on cell envelope integrity, LOS, or cell morphology, we carried out Etest antibiotic susceptibility experiments (S2 Table), isolated and stained LOS, and performed scanning electron microscopy studies. No differences were noted in LOS migration and abundance (Fig 4E), nor were the overall morphology or cell sizes altered in any gonococcal strain (Fig 4F). In addition, solely the **Δ***acp***Δ***sliC* mutant showed a two-fold decrease in minimal inhibitory concentration (MIC) towards benzylpenicillin, suggesting modest disruption in cell envelope homeostasis (S2 Table).

### SliC does not function as an adhesin

**Fig 5:**
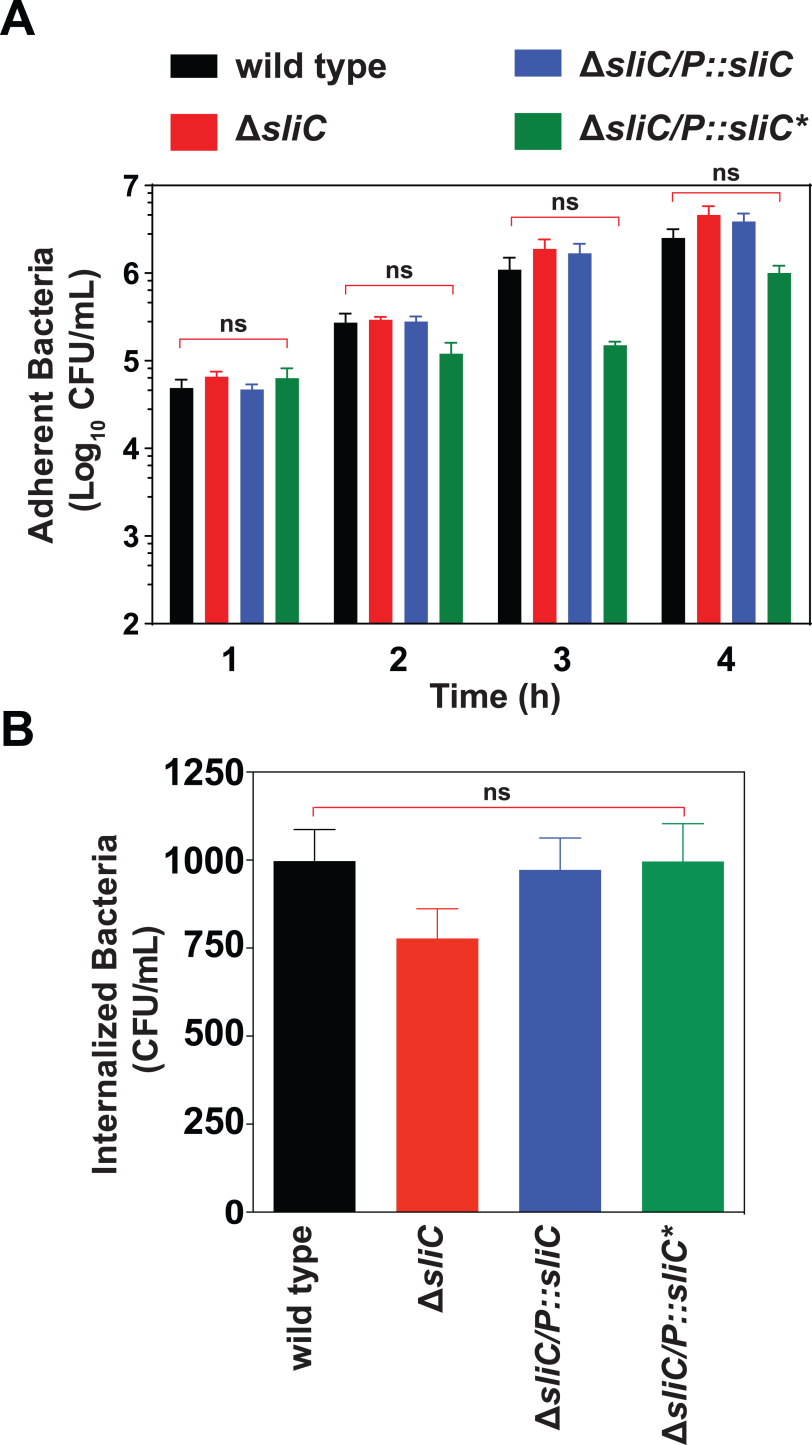
SliC does not function as an adhesin. (A, B) *N*. *gonorrhoeae* FA1090 wild type and isogenic Δ*sliC*, Δ*sliC*/*P*::*sliC*, and Δ*sliC*/*P*::sliC* were grown for 18 h on solid media, suspended, and added to human cervical epidermal carcinoma (ME180) cells at a MOI of 10:1 for 1, 2, 3, and 4 h. (A) For adherence experiments, cells were washed with PBS and treated with 1% saponin for 15 min. Lysates were collected and aliquots plated on solid media. Results are expressed as the number of CFUs recovered [total number of cell-associated bacteria; (*n* = 6; mean ±SEMs] (B) For invasion assays, human cells infected with each strain were treated with 100 μg/mL of gentamicin for 2 h. After washing to remove adherent bacteria, the number of invasive gonococci was quantified by lysing the cells with 1% saponin for 15 min. Aliquots of the lysates were plated on solid media. Results are expressed as the number of CFUs recovered after gentamycin treatment (*n* = 3; mean ±SEMs). The numbers of adherent and internalized bacteria between analyzed strains and wild type *N*. *gonorrhoeae* were not statistically significant (ns).

Surface-exposed lipoproteins are linked to a wide range of roles including bacterial adhesion [22, 28]. In *Neisseria*, the lipoprotein heparin-binding antigen (NHBA) facilitates adhesion to the surface of human epithelial cells [29] and ACP functions as both a lysozyme inhibitor and an adhesin in *N*. *meningitidis* [9, 30]. Therefore, to examine whether SliC plays a similar dual function in *N*. *gonorrhoeae*, we performed time-course adhesion experiments and invasion assays with human cervical epidermal carcinoma ME180 cells *in vitro*. Similar numbers of adherent (Fig 5A) and internalized (Fig 5B) gonococci were recovered for wild type, Δ*sliC*, and both complemented strains at all timepoints, demonstrating that SliC does not contribute to bacterial association with and invasion to human cervical ME180 cells.

### SliC is expressed throughout bacterial growth *in vitro* and during lower genital tract infection of female mice

**Fig 6:**
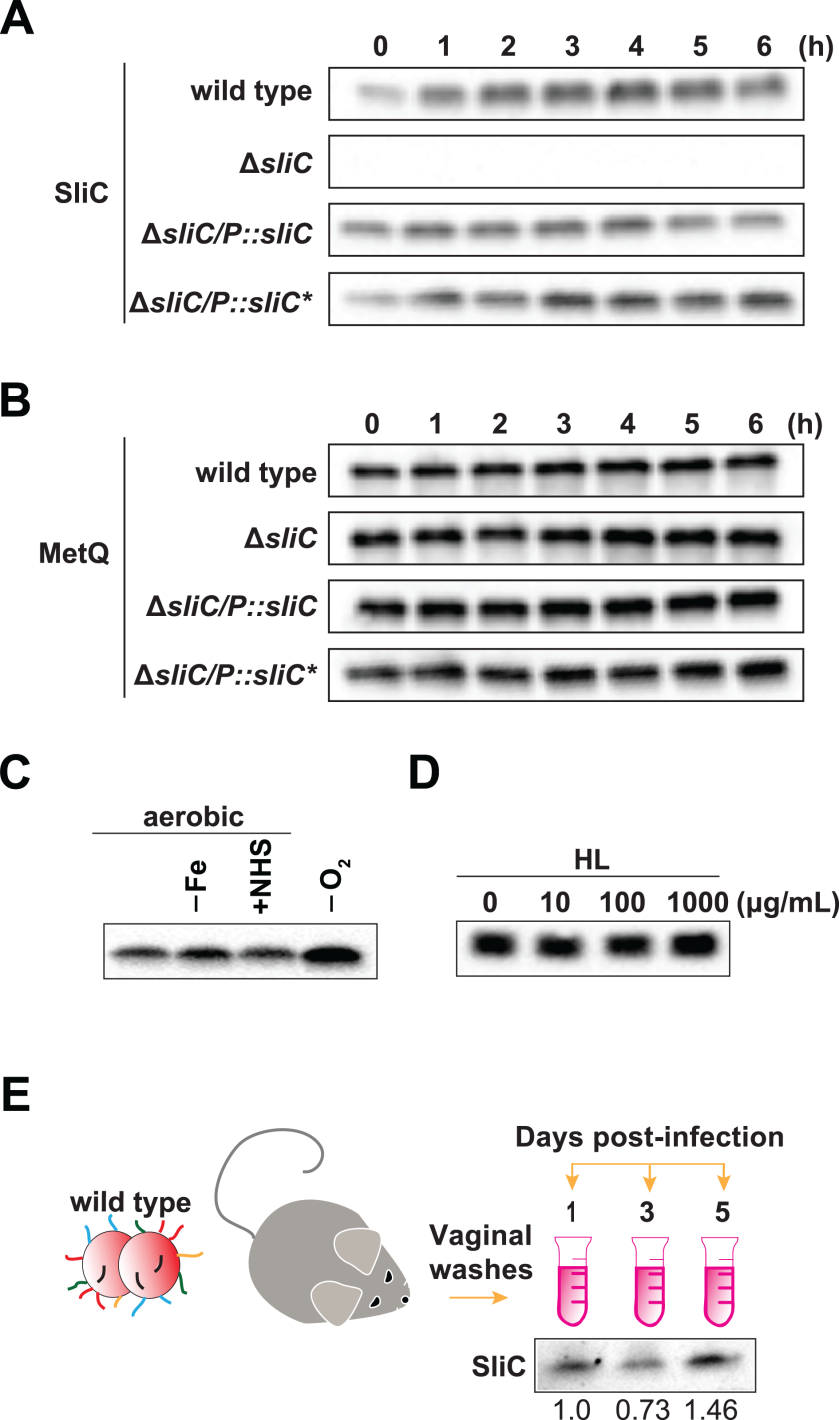
Expression patterns of SliC *in vitro* and *in vivo*. (A-B) Expression of SliC (A) and MetQ (B) was examined in the wild type, Δ*sliC*, **Δ***sliC/P*::*sliC*, and **Δ***sliC*/*P*::sliC* of N. *gonorrhoeae* FA1090 throughout growth in liquid media. At the time points indicated, samples were withdrawn and processed for SDS-PAGE and immunoblotting. (C) Quantities of SliC in wild‐type FA1090 during *in vitro* conditions relevant to different micro-infection sites [standard growth under aerobic conditions on solid media (SGC), iron deprivation (‐Fe), presence of 7.5% normal human serum (+NHS), and anaerobiosis (‐O_2_)] were assessed by probing the whole cell lysates with respective antibodies. (D) Expression of SliC was examined in whole-cell extracts derived from wild type FA1090 incubated for 3 h at 37° C with increasing concentrations of human lysozyme (0, 10, 100, and 1000 μg/mL) and lactoferrin (3 mg/mL). (E) Groups of BALB/C mice were inoculated intra-vaginally with 10^6^ CFUs of wild type FA1090. Vaginal swabs were collected on days 1, 3, and 5 post-inoculation and suspended in 100 μL of liquid media. Serial dilutions of vaginal swabs were cultured quantitatively on agar with streptomycin to enumerate the recovered CFUs while aliquots of the same suspensions were prepared for SDS-PAGE by the addition of lysis buffer. For experiments shown in panels A-D, samples containing the whole-cell lysates were matched by equivalent OD_600_ units; for experiments reported in panel E, samples were matched by the same CFUs. All samples were resolved by SDS-PAGE and transferred onto nitrocellulose. Immunoblots were performed using polyclonal rabbit antisera against SliC (A-E) and MetQ (B). SliC abundance relative to day 1 postinfection, determined by densitometry analysis, is denoted below the immunoblot.

To further assess SliC function in *N*. *gonorrhoeae* biology, expression of the protein was examined *in vitro* and *in vivo* during colonization of the female mouse genital tract (Fig 6). These studies showed that gonococci produce SliC throughout different growth phases in liquid media, similar to the ubiquitously-expressed MetQ [16], which was used as a comparison (Fig 6A and 6B). SliC was also expressed at comparable levels under different host-relevant conditions, such as iron deprivation and in the presence of normal human sera. Interestingly, an increase in the SliC cellular pool was observed during anaerobic growth (Fig 6C).

The *E*. *coli* MliC and Ivy proteins are both part of the Rcs extra-cytoplasmic stress regulon that responds to lysozyme insult by increased expression [31]. We wondered whether *sliC* induction could result from exposure to lysozyme. To test this hypothesis, wild type *N*. *gonorrhoeae* was incubated in the presence of lactoferrin and increasing concentrations of HL. No significant changes in the SliC cellular pool between untreated and lysozyme-treated cells were revealed by immunoblot analysis (Fig 6D).

Different members of the MliC/PliC family have been implicated in bacterial pathogenesis [8]. However, studies which demonstrate their expression *in vivo* are lacking to date. Therefore, we next sought to examine expression of SliC during *N*. *gonorrhoeae* colonization of the female mouse vaginal tract. Female BALB/c mice were infected with wild type FA1090 and vaginal washes were collected at days 1, 3, and 5 post-infection and pooled. Equal numbers of bacterial cells (CFUs) were separated by SDS-PAGE and probed with anti-SliC antisera. These studies showed that SliC was readily produced throughout the 5-day infection period (Fig 6E). Densitometry analyses using SliC abundance on day 1 as a reference showed that the amounts of SliC lowered to 0.73 fold on day 3 postinfection and increased to 1.46 fold on day 5 when compared to the amount of SliC detected on day 1.

Cumulatively, these experiments demonstrated that *N*. *gonorrhoeae* stably produces SliC during different growth conditions *in vitro* and provide the first evidence for the expression of a MliC/PliC protein *in vivo*, suggesting that maintenance of this lysozyme c inhibitor is important during infection.

### SliC provides a significant survival advantage for *N*. *gonorrhoeae* during mucosal infection

To test the potential significance of SliC in gonococcal virulence, we performed preliminary *in vivo* competitive infection experiments with the **Δ***sliC* mutant and the wild type parent strain in the female mouse model of gonococcal genital tract infection. In two independent experiments, the lack of SliC caused a dramatic 10, 250, and 167-fold attenuation of *N*. *gonorrhoeae* colonization on days 1, 3, and 5 post-infection, respectively (S3 Fig).

**Fig 7:**
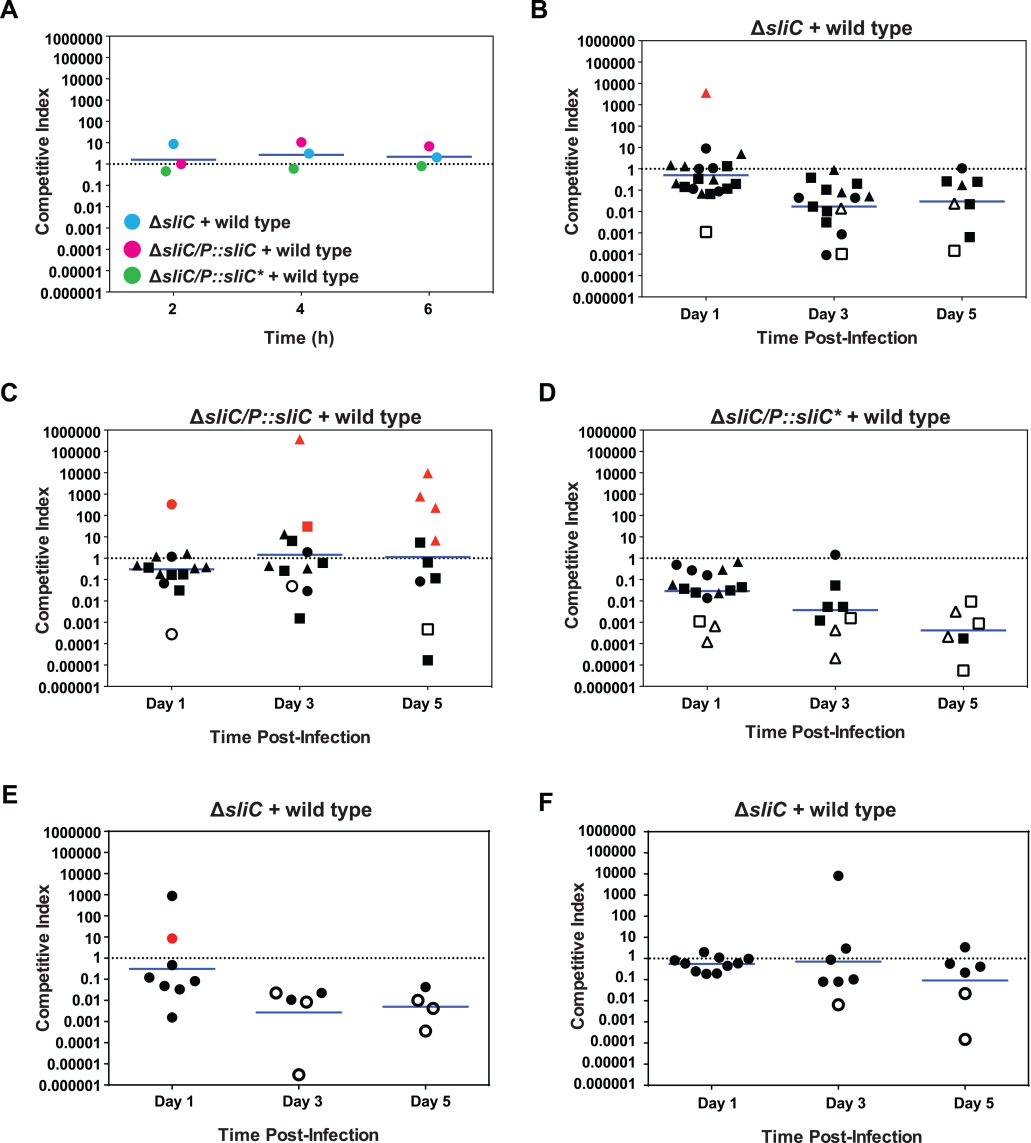
SliC provides a significant survival advantage for *N*. *gonorrhoeae* during mucosal infection and the virulence defect is dependent on the lysozyme inhibition function. (A) *In vitro* competition assays were executed in liquid media concurrently with three sets of strains: wild type and Δ*sliC*, wild type and **Δ***sliC/P*::*sliC*, or wild type and **Δ***sliC*/*P*::sliC*. Equivalent CFUs (10^6^ CFUs) of each strain were inoculated into liquid media and the CFU output was assessed at 2, 4, and 6 h post-inoculation. (B-D) Groups of BALB/c mice were inoculated intravaginally with wild-type FA1090 combined with similar CFUs (total dose, 10^6^ CFU *N*. *gonorrhoeae*; 7 mice/group) of isogenic Δ*sliC (B)*, **Δ***sliC*/*P*::*sliC* (C), or **Δ***sliC*/*P*::sliC* (D). Vaginal swabs were collected on days 1, 3, and 5 post-inoculation and suspended in liquid media. Vaginal swab suspensions and inocula were cultured quantitatively on agar with streptomycin (total number of CFUs) and solid media with streptomycin and kanamycin to enumerate Δ*sliC*, **Δ***sliC*/P::*sliC*, or **Δ***sliC*/P::sliC*. Results are expressed as the competitive index (CI) using the equation CI = [mutant CFU (output)/wild-type CFU (output)]/[mutant CFU (input)/wild-type CFU (input)]. The limit of detection of 1 CFU was assigned for a strain that was not recovered from an infected mouse. A CI of <1 indicates that the mutant is less fit than the wild type strain. All experiments were performed in biological triplicates and geometric means are shown. Statistical analysis was performed using Kruskal-Wallis Dunn’s multiple comparison tests to compare statistical significance of CIs between the **Δ***sliC/*wild type and **Δ***sliC*/*P*::*sliC/*wild type as well as **Δ***sliC*/*P*::sliC*/wild type and **Δ***sliC*/*P*::*sliC/*wild type. (E-F) Competitive infections were also performed with mixtures of wild type FA1090 and similar numbers of isogenic Δ*sliC* in C57BL/6J (E) and B6.129P2-Lyz2tm1(cre)Ifo/J 9 mice (F), which do not produce lysozyme. Results from two experiments were combined. Statistical analysis was performed using Mann-Whitney test to compare statistical significance of CIs between the Δ*sliC*/wild type in C57BL/6J and Δ*sliC*/wild type in lysozyme knockout mice. For all experiments, red symbols indicate mice from which no wild type bacteria were recovered, while open symbols designate no mutant CFUs were recovered.

We next conducted competitive infection experiments in which the **Δ***sliC* mutant and the **Δ***sliC*/*P*::*sliC* and **Δ***sliC*/*P*::sliC* complemented mutants were competed with the wild type strain in parallel. The competitive indices obtained for all strains did not differ during *in vitro* co-culture (Fig 7A), whereas results from three independent coinfection experiments demonstrated a severe reduction in the relative number of CFUs recovered from vaginal swabs for the **Δ***sliC* knockout and **Δ***sliC*/*P*::sliC* mutant (Fig 7B–7D, respectively). The recovery of the Δ*sliC* mutant was approximately 2-, 58-, and 34-fold (geometric mean CIs) lower than the wild type strain on days 1, 3, and 5 post-inoculation, respectively (Fig 7B). The fitness disadvantage was exacerbated for the **Δ***sliC*/*P*::sliC* bacteria, with averages of 34, 270, and 2,375-fold lower than for wild type gonococci on days 1, 3, and 5 post-infection, respectively (Fig 7D). In contrast, the defect was fully restored by genetic complementation in the **Δ***sliC*/*P*::*sliC* strain (Fig 7C). When Kruskal-Wallis Dunn’s multiple comparison tests were performed to compare statistical significance of CIs between the **Δ***sliC*/wild type and **Δ***sliC*/*P*::*sliC*/wild type, the values were >0.99, 0.027, and 0.77 on days 1, 3, and 5 post-inoculation. The calculated *p* values for the **Δ***sliC*/*P*::sliC*/wild type versus **Δ***sliC*/*P*::*sliC*/wild type were all significant and reached 0.029, 0.0058, and 0.016.

To test the basis for the attenuation of Δ*sliC* bacteria relative to wild type, we next conducted competitive infections in mice that were genetically defective for lysozyme using C57BL/6J mice as the background controls. Data from two combined experiments showed that the relative recovery of the Δ*sliC* mutant from C57/BL6J mice was approximately 3-, 372-, and 198-fold lower (geometric mean CIs) than the wild type strain on days 1, 3, and 5 post-inoculation, respectively (Fig 7E). In contrast, the Δ*sliC* mutant was recovered at the same level as the wild type strain in mice that lack lysozyme on days 1 and 3, while on day 5 the geometric mean CI was 10-fold lower than the wild type strain (Fig 7F). The difference in the CIs in C57BL/6J mice and lysozyme knockout mice was statistically different on day 3 (*p* value of 0.03).

Only two reports to date examined the *in vivo* role of lysozyme inhibitors in bacterial virulence [13, 14] but it has never been dissected whether the virulence defects observed were dependent on the lysozyme inhibition function. Together these studies demonstrated that SliC provides a significant fitness benefit to *N*. *gonorrhoeae* in the murine model and offered the first evidence that a proteinaceous lysozyme c inhibitor is important during *in vivo* infection solely due to its ability to bind and inactivate lysozyme.

## Discussion

To establish infection, microbes must evade a combination of host antimicrobial peptides and enzymes, including lysozymes, which are abundantly secreted by the epithelium and produced within professional phagocytes [32]. Described for the first time by Alexander Fleming in 1922 as a substance with the ability to “lyse” bacteria, lysozyme additionally acts as an antimicrobial peptide by interacting directly with cell membranes via its positively charged amino acids [33]. Pathogenic and commensal bacteria have developed many methods to evade lysozyme; however, until the recently reported ACP [9], no proteinaceous lysozyme inhibitor has been described for the genus *Neisseria*. ACP is a surface-exposed meningococcal adhesin that induces cross-strain bactericidal antibodies [30]. Despite low primary sequence homology, the *apo* crystal structure of *N*. *meningitidis* ACP resembles an eight-stranded antiparallel ß-barrel similar in the overall fold to those of MliC/PliC proteins [9, 11, 12, 19]. *In vitro* assays have demonstrated that ACP proteins act as inhibitors of HEWL and HL, contributing to lysozyme tolerance in the presence of the membrane-permeabilizing agent lactoferrin [9, 30]. Nevertheless, the importance of lysozyme inhibition for host colonization has not been addressed in *Neisseria* and investigations in other bacterial species are also sparse [13, 14].

In this report, we demonstrated for the first time that inhibition of lysozyme activity is pivotal for *N*. *gonorrhoeae* mucosal infection in the female genital tract. Through genetic, biochemical, *in vitro* and *in vivo* functional assays, we characterized a previously unrecognized surface-exposed protein, SliC, that plays a role in virulence as a weapon to counteract c-type lysozyme and contributes to *N*. *gonorrhoeae* colonization. We originally identified SliC (NGO1063) as a potential vaccine/drug candidate in quantitative proteomic surveys of the *N*. *gonorrhoeae* cell envelopes and naturally released membrane vesicles [15, 16].

We found that SliC belongs to the MliC/PliC family of lysozyme inhibitors (Fig 1) and is actually closer to subgroup 1 MliCs, represented by *E*. *coli* MliC, than to subgroup 2 MliCs, represented by *P*. *aeruginosa* MliC (Fig 1A). SliC possesses the FWSKG motif commonly present in subgroup 1 MliCs and does not have any of the conserved hydrophobic residues characteristic of subgroup 2 [10, 14]. Our phylogenetic analysis, however, demonstrated that *Neisseria* SliC and ACP, despite their low primary amino acid sequence identity (S1 Table), were more closely related to each other than either protein was to MliC/PliC lysozyme inhibitors from other bacteria (Fig 1B).

MliC proteins are found anchored to the periplasmic side of the outer membrane, whereas PliC family members are localized in the periplasm [8]. In our proteomic investigations, SliC was found in cell envelopes and naturally released membrane vesicles [15]. We confirmed these initial observations by performing sub-cellular fractionation experiments coupled with immunoblotting using antisera specific to SliC as well as control protein markers (Fig 2D). In addition, we showed by two independent approaches–protease accessibility studies (Fig 2E) and immunodotting (Fig 2F)–that SliC is a surface-displayed lipoprotein, which we predicted earlier with *in silico* approaches [15]. Similarly, the *Mycobacterium tuberculosis* lysozyme inhibitor, LprI, is a surface-exposed lipoprotein [34]. Surface-localization of ACP and SliC appears to be another smart strategy employed by *Neisseria* to guard against the devastating action of lysozyme before it traverses the outer membrane, which is facilitated by the permeabilizing action of other host antimicrobial peptides. The conservation and expression of SliC throughout phases of growth, in conditions relevant to different micro-niches of the human host, during experimental infection of female mice, and among diverse gonococcal isolates, as well as in *N*. *meningitidis*, *N*. *lactamica*, and *N*. *weaveri*, further highlights the importance of maintaining this surface-exposed outer membrane protein in pathogenic, commensal and opportunistic *Neisseria* (Figs 1C–1F, 6 and S1–S2).

To better understand the mechanism of lysozyme inhibition, different biochemical approaches were utilized for several members of the MliC/PliC family and for ACP [8, 9, 11, 12, 14, 18, 19, 34]. Our studies with untagged rSliC demonstrated that SliC inhibits both HEWL and HL (Fig 3A and 3B) but is a more potent inhibitor of HL (Fig 3C) with a calculated IC_50_ of 1.85 μM. Similar observations have been made for *N*. *meningitidis* ACP [9]. Site-directed mutagenesis of the two residues in SliC corresponding to the sites of *P*. *aeruginosa* MliC interaction with HEWL [11], S83 and K103, led to complete abrogation of lysozyme hydrolytic activity (Fig 3D), suggesting that SliC employs a similar mode of lysozyme inhibition as MliC proteins. To further evaluate the SliC-HL interaction, BLI was utilized, which yielded a calculated K_D_ value of 9.2 ± 1.9 μM (average ± SEM; Fig 3F). This result suggests a moderate binding of SliC to HL *in vitro*, which is very similar to the ACP-HL interaction, with a K_D_ of 11 μM. Intriguingly, MliC from *Brucella abortus* shows a thousand-fold higher binding affinity [12]. It is likely that SliC and ACP lysozyme binding affinities are influenced by the presence of peptidoglycan, the lysozyme substrate.

Our complementary biochemical approaches demonstrated that SliC is an inhibitor of c-type lysozyme (Fig 3); however, exposure of *N*. *gonorrhoeae* to HL did not result in any significant differences in bacterial viability in comparison to the wild type strain (Fig 4C). We concluded that other mechanisms employed by *N*. *gonorrhoeae* to resist killing by lysozyme, ACP and lytic transglycosylases, for example [9, 26, 27], compensated for the lack of SliC and were sufficient to provide resistance *in vitro*. Deletion of *acp* did not render the bacteria more sensitive to lysozyme (Fig 4C), which was in contrast to an earlier observation [9, 26, 27] perhaps due to differences between gonococcal isolates. It is plausible that a positive feedback loop exists between ACP and SliC that provides increased levels of ACP upon decreases in SliC expression. Supporting this conclusion, removal of both SliC and ACP rendered the bacteria remarkably sensitive to lysozyme, which was entirely complemented in the Δ*acp* Δ*sliC*/*P*::*acp P*::*sliC* strain (Fig 4C).

Most importantly, SliC and its lysozyme inhibition function conferred a fitness advantage to *N*. *gonorrhoeae in vivo*, which we demonstrated in preliminary competition experiments with the **Δ***sliC* mutant against wild type during co-infection in the murine female genital tract (S3 Fig) and in biological triplicate studies (Fig 7B–7D), in which the **Δ***sliC*/*P*::sliC* mutant strain, in addition to **Δ***sliC* and the complemented strain, **Δ***sliC*/P::*sliC*, were challenged against equal numbers of wild type bacteria. A drastic decrease in **Δ***sliC* fitness *in vivo* was observed (Fig 7B), which was further exacerbated for the **Δ***sliC*/*P*::sliC* bacteria, which expresses a functionally inactivated SliC protein (Fig 7D). This statistically significant drop in fitness was not caused by altered LOS (Fig 4E), cell morphology (Fig 4F), or other potential defects in the outer membrane integrity in cells lacking SliC, as no significant differences were observed between wild type and Δ*sliC* bacteria in sensitivity to seven antibacterial compounds with different modes of action (S2 Table). Further, bacterial fitness could be fully restored by genetic complementation in the **Δ***sliC*/*P*::*sliC* strain (Fig 7C). Nor was the defect triggered by instability or affected transport of SliC* to the gonococcal cell surface, as the mutated protein was stably expressed from its native promoter (Fig 2C) and efficiently escorted to the gonococcal cell surface (Fig 2F). No significant changes in the numbers of adherent and internalized bacteria were found in our *in vitro* studies (Fig 5) eliminating SliC as an adhesin. Finally, our studies involving lysozyme defective mice (Fig 7E and 7F) further support SliC as a critical inhibitor of lysozyme during infection. Lysozyme is detected in the human cervico-vaginal fluid proteome and may be of either phagocytic or epithelial origin [35–38].

Taken together, our studies provide the first evidence that a novel lysozyme-binding lipoprotein of *N*. *gonorrhoeae*, SliC, plays a critical role during colonization of the host. Lysozyme is a major host defense factor against bacteria. Therefore, a detailed understanding of the mechanisms that bacteria have developed to fight lysozyme may allow development of new antimicrobial strategies against pathogens and also against certain microbiota during dysbiosis.

## Materials and methods

### Bacterial strains and growth conditions

*Neisseria* utilized in this study include *N*. *gonorrhoeae* FA1090 [39] and 36 temporally and geographically diverse gonococcal clinical isolates with the 2016 WHO *N*. *gonorrhoeae* reference strains [16, 40], *N*. *meningitidis* MC58 [41], *N*. *lactamica* NL1983/-01, and *N*. *weaveri* 1032 [42]. *Neisseria* were removed from frozen stocks and plated on gonococcal base solid medium (GCB, Difco) and incubated in a 5% CO_2_ atmosphere at 37°C for 18–20 h. Transparent, non-piliated colonies were subcultured onto GCB. After passage on GCB, bacteria were cultured in gonococcal base liquid (GCBL) medium supplemented with Kellogg’s supplement I in 1:100 and 12.5 μM ferric nitrate [43, 44]. To achieve iron-limited conditions, GCB without ferric nitrate and with deferoxamine mesylate salt (Desferal, Sigma) at 5 μM final concentration was utilized [45]. In addition, when stated in the text, GCB supplemented with 7.5% normal human serum [46] was used. For anaerobic growth, bacteria were plated on GCB with 1.2 μM nitrite as a terminal electron acceptor and harvested after 48 h [16]. Except where otherwise indicated, colonies were collected from plates with a polyester-tipped sterile applicator (Puritan) and suspended to an OD_600_ of 0.1 in GCBL supplemented with 0.042% sodium bicarbonate and supplements as above [43, 44]. Liquid cultures were propagated at 37°C with shaking for 3 h, back-diluted to an OD_600_ of 0.1 in supplemented GCBL, and cultured in the same manner. Piliated colonies were used for transformation and adhesion/invasion assays while non-piliated variants were utilized in all other experiments.

*Escherichia coli* strains were grown either on Luria-Bertani agar (LBA, Difco) or cultured in Luria-Bertani broth (LB, Difco) at 37°C.

Antibiotics were used in the following concentrations: for *N*. *gonorrhoeae*: kanamycin 40 μg/mL, erythromycin 0.5 μg/mL, chloramphenicol 0.5 μg/mL, streptomycin 100 μg/mL; for *E*. *coli*: kanamycin 50 μg/mL, carbenicillin 50 μg/mL, chloramphenicol 34 μg/mL.

### Genetic manipulations and site directed mutagenesis

Cloning procedures were performed in *E*. *coli* MC1061 [47]. *N*. *gonorrhoeae* FA1090 (NC_002946) genomic DNA was isolated using the Wizard Genomic DNA Purification Kit (Promega). Oligonucleotides were designed using SnapGene software version 2.8 (GSL Biotech LLC) and synthesized by Integrated DNA Technologies. Q5 High-Fidelity DNA polymerase, DNA ligase and NEBuilder HiFi DNA Assembly Master Mix were purchased from New England Biolabs (NEB). Site-directed mutagenesis was performed using Q5 Site-Directed Mutagenesis Kit (NEB). All obtained genetic constructs were verified by Sanger Sequencing at the Center for Genomic Research and Biocomputing at Oregon State University. Transformation of *N*. *gonorrhoeae* was performed as described previously [48].

To create an in-frame knockout of *sliC*, individual 500 bp fragments upstream (sliC_up) and downstream (sliC_down) of *sliC* were amplified using primer pairs 5’ACGTTGAGAATTCGCCGTCTGAGTCGGAATATGTCGGAGC3’, 5’AGCGTACAGGTACCAGCGCGAAAAACCTGATA3’, and 5’ACTCAATAGGATCCCGAAACTTCCTGCCGC3’, 5’ACTCGGTCAAGCTTCATTGGATACCGACAATGAAAC3’. The obtained sliC_up and sliC_down PCR products were digested with EcoRI/KpnI and BamHI/HindIII, respectively. Subsequently, sliC_up was cloned into similarly digested pUC18K, yielding pUC18K-upsliC and sliC_down was cloned into this genetic construct to yield pUC18K-ΔsliC. This plasmid was digested with ScaI and used for liquid transformation of *N*. *gonorrhoeae* FA1090 [48]. The *sliC* knockout was verified using primers 5'GGTTGGCGATGTAGAGGCT3’ and 5'GATTGCAGTTACAACGCGTGG3' and chromosomal DNA isolated from wild type FA1090 as controls, as well as immunoblotting analysis with anti-SliC antisera of the whole cell lysates obtained from wild type and Δ*sliC*.

An in frame and markerless knockout of *acp* was achieved using Gibson assembly method [49]. Briefly, pNEB193 and DNA fragments comprising 1000 bp upstream and downstream from *acp* gene were amplified using primers: 5’GTTTAAACCTGCAGGCATGCAAG3’, 5’TCTAGACTTAATTAAGGATCCGGCG3’, 5’CGCCGGATCCTTAATTAAGTCTAGAACAAGCCGCTCAAAGAAGGTGACATTATCAACATC3’, 5’ATTATTCAGACGGCATTTACGGCGGCCTGTCCGGTGT3’, 5’GACAGGCCGCCGTAAATGCCGTCTGAATAATCAGGCAACAAAAAACAGCGTTTTCATTTC3’, 5’AGCTTGCATGCCTGCAGGTTTAAACCGCCGATACCCGCAACCC5’ and Q5 polymerase. The obtained DNA fragments were purified and assembled using Hi-Fi Assembly Mix (NEB) to create pNEB-Δacp plasmid. This plasmid was linearized with ScaI and used for spot transformation of *N*. *gonorrhoeae* FA1090. The FA1090 Δ*acp* knockout was confirmed by PCR reaction using primers 5’GACCGGGATGAACCAGATAG3’ and 5’GGCTGATGCACCAATGCTTC3’.

The FA1090 Δ*acp* knockout strain and pUC18K-ΔsliC were used to create the isogenic Δ*acp* Δ*sliC* double mutant. Briefly, pUC18K-ΔsliC was cut with ScaI and used for spot transformation of *N*. *gonorrhoeae* FA1090 Δ*acp*. The deletion of *sliC* in Δ*acp* Δ*sliC* double knockout was confirmed by PCR with the primer pair: 5'GGTTGGCGATGTAGAGGCT3’ and 5'GATTGCAGTTACAACGCGTGG3', as well as by probing whole cell lysates obtained from wild type and Δ*acp*Δ*sliC* in immunoblotting analysis with anti-SliC antisera.

Constructs for complementation of Δ*sliC* with the wild type *sliC* allele or sliC* (carrying alanine substitutions in S83 and K103) and for production of recombinant proteins, rSliC and rSliC*, were designed as outlined in Fig 2A using the following steps. First, *sliC* was amplified with its native promoter using primers 5’GATCTTAATTAATTCAGACGGCATCGTCAGGC3’ and 5’GATCGTTTAAACTCAAACAGGCTGCCCCGT3’. The resulting PCR product was digested with PacI/PmeI and cloned into similarly treated pNEB193, yielding pNEB-sliC. To create sliC*, the S83A substitution was introduced with primers 5’CGTTGCCGCAGCTGGCGAACGCT3’ and 5’TCGGAAGAGAGGACGGCACG3’ using pNEB-sliC as template. The K103A mutation was generated in SliC S83A using primers 5’GTGGCACCAGGCTGGCGGCGAAG3’ and 5’TCGGTTCCGTTTCCGAAC3’.

To create constructs for complementation of Δ*sliC*, pNEB-*sliC* and pNEB-sliC* were treated with PacI/PmeI and DNA fragments corresponding to *sliC* and sliC*, respectively, were cloned into similarly digested pGCC5 [50]. The obtained plasmids, pGCC5-*sliC* and pGCC5-sliC*, were introduced to FA1090 Δ*sliC*. The presence of the integrated recombinant genes was confirmed in transformants by PCR, and the level of SliC expression was confirmed by immunoblotting with anti-SliC antisera.

To complement the Δ*acp* mutant, the *acp* gene with its native promoter was PCR amplified using primers 5’GATCGTCGACCCATATCGAATGCCTCGA3’ and 5’GATCTTAATTAAGGAACGGTCAAAAAACAGC3’. The resulting DNA fragment was digested with PacI and cloned into PacI/PmeI digested pGCC5 plasmid to yield pGCC5-acp. This plasmid was introduced into FA1090 Δ*acp* by transformation and the presence of the integrated *acp* was confirmed by PCR.

To complement the FA1090 Δ*acp*Δ*sliC* double knockout strain, pGCC5-acpsliC plasmid carrying both genes under control of their native promoters was constructed as follows: *acp* gene and 240 bp upstream from its start codon was amplified by PCR using the primer pair 5’GATCGTCGACCCATATCGAATGCCTCGA3’ and 5’GATCTTAATTAAGGAACGGTCAAAAAACAGC3’. The resulting DNA fragment was digested with SalI/PacI and cloned into similarly cut pGCC5-sliC to yield pGCC5-acpsliC used for spot transformation of FA1090 Δ*acp*Δ*sliC*. The presence of *acp* and *sliC* alleles in heterologous location was subsequently confirmed by PCR and their functionality was verified by immunoblotting and complementation studies.

Constructs used for production of recombinant wild type SliC (rSliC) and SliC bearing S83A K103A substitutions (rSliC*), both lacking a signal peptide and containing a C-terminal Tobacco Etch Virus (TEV) protease cleavage site followed by a 6×His-tag, were created by amplifying *sliC* and sliC* with primers 5’CGTAATGCCATGGTGCCGGAAGCGTATGATGGC3’ and 5’AGTCAGCAAGCTTACGGGCGCGGCAGG3’ using pNEB-sliC and pNEB-sliC* as templates, respectively. The obtained PCR products were digested with NcoI/HindIII and cloned into pRSF-NT [51].

### Protein purification

Recombinant SliC and SliC* were purified from 3 L cultures of *E*. *coli* BL21(DE3) [52] carrying pRSF-rSliC and pRSF-rSliC*, respectively. Bacterial cultures were incubated at 37°C until OD_600_ of 0.7 and induced with 0.5 mM IPTG. The cultures were incubated overnight at 16°C and the cells were pelleted at 8,000 × *g* for 15 min. Cells were suspended in lysis buffer (20 mM HEPES pH 7.5, 500 mM NaCl, 10 mM imidazole) and lysed by passing four times through French Press at 1,500 psi. Cell debris was removed by centrifugation at 17,000 × *g* for 20 min and the cleared lysate was applied to a 5 mL IMAC column (BioRad). Proteins were purified using a NGC Medium-Pressure Liquid Chromatography System (BioRad) using lysis buffer and elution buffer (20 mM HEPES pH 7.5, 500 mM NaCl, 250 mM imidazole). Fractions containing either rSliC or rSliC* were pooled and the buffer was supplemented with DTT and EDTA to final concentrations of 1mM and 0.5 mM, respectively. The His-tag was removed by overnight incubation at 4°C with TEV protease in a 1:25 ratio. The samples were concentrated using a Vivaspin 20 centrifuge concentrator (GE HealthCare) and subjected to size exclusion chromatography using a HiLoad 16/600 Superdex 75 pg column (GE HealthCare) with phosphate buffered saline (PBS) as running buffer to separate SliC and SliC* from TEV protease. Protein purity was confirmed by SDS PAGE and the protein concentration was measured using the BioRad DC Protein Assay Kit.

### Antibody generation

Polyclonal antisera against purified rSliC were prepared by Pacific Immunology Corp. using a 13-week antibody production protocol and two New Zealand White rabbits under Animal Protocol #1 approved by IACUC and the NIH Animal Welfare Assurance Program (#A4182-01) in a certified animal facility (USDA 93-R-283).

### Lysozyme activity assay

The EnzChek Lysozyme Assay kit (ThermoFisher) was used to determine the SliC-mediated inhibition of c-type lysozyme from chicken egg white (HEWL, Sigma) and human lysozyme (HL, Sigma). Assays were conducted using 96 well plates and 100 μL volumes of 0.1 M sodium phosphate, 0.1 M NaCl, pH 7.5 and 2 mM sodium azide as buffer. Experimental samples containing 2.5 μM of either HEWL or HL were incubated with increasing concentrations of SliC (0–10 μM) for 30 min at 37°C. The control wells contained HEWL or HL alone. After incubation, the reaction was initiated by addition of 50 μL of the 50 μg/mL DQ lysozyme substrate. The reaction was monitored for 1 h using a Synergy HT Microplate Reader (BioTek) at excitation and emission wavelengths of 485 nm and 530 nm, respectively. To calculate the IC_50_, results were exported to GraphPad Prism 6 (GraphPad Software). To test the inhibition of HL by SliC*, 2.5 μM of HL and 5 μM of the protein was used.

Comparison of SliC inhibition of chicken and human lysozyme was assessed by incubation of 2.5 μM final lysozyme solution with increasing concentrations of SliC (0–10 μM). After mixing the proteins, the assay was performed as described above.

### BioLayer interferometry

The binding affinity of SliC to lysozyme was assessed by BLI on an OctetRed 96 (ForteBio). The SliC protein was randomly biotinylated on surface-exposed lysine side chains with succinimidyl-6-(biotinamido)hexanoate, a biotin label with a medium chain length spacer arm, per manufacturer’s instructions (EZ- Link NHS-LC-Biotin, Pierce). SliC was incubated with a 20-fold molar excess of biotin reagent 1 h at room temperature, and protein was subsequently applied to a PD10 column to remove excess biotin reagent. The SliC and HL samples were prepared in Kinetic Buffer (20 mM HEPES pH 8, 15 mM NaCl, 0.002 Tween 20, 0.1 mg/mL BSA). Streptavidin (SA) biosensors (ForteBio) were loaded with biotinylated SliC for 10 min at a 20 μg/mL concentration. Unloaded tips were used as a control. The baseline was established for 240 s and the association and dissociation steps were performed for 600 s. Experiments were performed in three biological replicates with curve fitting using a 2:1 (Heterogeneous Ligand) model. K_D_ value calculations were completed using Octet Data Analysis (version 9).

### Assessment of subcellular localization

Subcellular fractionation procedures, dot-blots and protease shaving assays were conducted as described previously [16]. Briefly, extraction of proteins from the cytosolic, cell envelope, membrane vesicle, and soluble supernatant fractions was performed using 500 mL cultures of wild type *N*. *gonorrhoeae* FA1090 at mid-logarithmic phase of growth. Cell envelope proteins were separated from cytoplasmic fraction by a sodium carbonate extraction procedure and differential centrifugation, while culture supernatants were subjected to filtration and ultracentrifugation to separate naturally-released membrane vesicles from soluble proteins. After centrifugation, the soluble protein fraction was precipitated at 4°C for 1 h using 15% trichloroacetic acid.

In experiments involving proteinase K shaving and immunodotting, intact bacterial cells were used [16]. For protease accessibility studies, *N*. *gonorrhoeae* FA1090 wild type was sub-cultured in GCBL for 3 h after collecting from solid media, diluted to OD_600_ of 0.1 and cultured until OD_600_ of ~ 1.0 was reached. Gonococci were gently centrifuged and suspended to OD_600_ of 2.0 in sterile PBS (pH 8.0) supplemented with 5 mM MgCl_2_. Bacterial suspensions (500 μL) were incubated for 1 h at 37°C with proteinase K at final concentrations of 0, 20, or 40 μg/mL. To deactivate protease, 10 μL of 50 mM phenylmethylsulfonyl fluoride (PMSF) was added. Bacteria were then washed with GCBL, subjected to SDS-PAGE, and probed with polyclonal antisera to determine protease susceptibility.

For immunodotting, *N*. *gonorrhoeae* strains were suspended in GCBL to an OD_600_ of 0.1, cultured under standard growth conditions for 3 h, harvested, and spotted as 5 μL suspensions onto nitrocellulose membranes after adjusting the OD_600_ to 2.0. The samples were dried at room temperature for 15 min and subjected to immunoblotting as described below. For assessing the expression of SliC during experimental murine infection, five mice were infected vaginally with strain FA1090 and vaginal washes were collected on days 1, 3 and 5 post-bacterial inoculation by pipetting 40 μl of sterile PBS in and out of the vaginas. This procedure was repeated three times for each mouse, and the resultant fluids from each time point were pooled.

### Lysozyme-lactoferrin treatment assays

Non piliated cells of wild type FA1090, Δ*sliC*, Δ*sliC* complemented with *sliC*, Δ*acp*, Δ*acp* complemented with *acp*, Δ*sliC* Δ*acp*, and Δ*sliC* Δ*acp* complemented with *sliC* and *acp* were suspended in GCBL with Kellogg’s supplements and 0.042% sodium bicarbonate to OD_600_ of 0.1 and cultured at 37°C for 3 h. Bacteria were diluted to 1×10^4^ CFU/mL and 170 μL of each culture was incubated for 8 h at 37°C with lactoferrin (5 mg/mL; InVitria) and increasing concentrations of human lysozyme (0, 1, 5, 10 and 20 mg/mL; BioVision) in a final volume of 200 μL. Bacterial suspensions were plated for CFUs scoring. All experiments were performed in 5 biological replicates and means with corresponding SEMs are presented.

To test if the presence of cell envelope permeating agents increases the lysozyme sensitivity of gonococci, Polymyxin B (100 U/mL), Tween 20 (0.001%), or bile salts (0.05%) reconstituted in GCBL were added alone or in combination, as indicated, to bacterial cell suspensions (1×10^4^ CFU/mL) containing lactoferrin (5 mg/mL) and lysozyme (5 mg/mL) as described above. We established in preliminary studies that to obtain viable bacteria, cultures can be incubated at 37° in the presence of all these antimicrobials up to 4 h. Accordingly, after that time period mixtures were serially diluted and spotted on GCB to enumerate CFUs. All experiments were performed in 3 independent trials and means with corresponding SEMs are presented.

### Fitness assessments under *in vitro* growth conditions

The growth kinetics of wild type *N*. *gonorrhoeae* FA1090 and isogenic Δ*sliC*, **Δ***sliC*/*P*::*sliC*, and **Δ***sliC*/*P*::sliC* mutants were conducted in GCBL under standard growth conditions. Bacteria were collected from GCB and suspended in GCBL to an OD_600_ of 0.1. Following 3 h of incubation at 37°C with aeration bacterial cultures were back-diluted to OD_600_ of 0.1 in fresh GCBL and cultured for additional 6 h. Samples were withdrawn for OD_600_ measurements and immunoblotting every hour (*n* = 4; mean ±SEMs).

To assess the viability of *N*. *gonorrhoeae* lacking *sliC* during host-relevant *in vitro* growth conditions, colonies of wild type FA1090 and Δ*sliC* were collected from GCB, suspended in GCBL to an OD_600_ of 0.1, and cultured for 3 h at 37°C with aeration. Subsequently, the cultures were normalized to OD_600_ = 0.2, serially diluted, and plated on solid media for standard growth conditions (SGC), iron limiting conditions, NHS, and anaerobic conditions, as described above. The CFUs were scored after 22 and 48 h for aerobic and anaerobic conditions, respectively (*n* = 3; mean ±SEMs).

### Electron microscopy

*N*. *gonorrhoeae* FA1090 wild type and isogenic Δ*sliC*, **Δ***sliC*/*P*::*sliC*, **Δ***acp*, and **Δ***acp*Δ*sliC* were incubated in GCBL until strains reached mid-logarithmic growth. Cells were pelleted at 4,000 × *g* for 3 min and washed twice with PBS filtered through 0.1 μm filter. Bacteria were suspended in PBS and 2.5 μL of the cell suspensions were spotted onto 300 mesh copper grids. Cells were allowed to attach to the grid for 15 min and excess PBS was removed. Gonococci were negatively stained using phosphotungstic acid. Images were acquired using a FEI Helios NanoLab 650 electron microscope at the Oregon State University Electron Microscopy Facility.

### Isolation of LOS and silver staining

LOS was isolated from *N*. *gonorrhoeae* as described previously [53]. Bacteria were collected from GCB plates incubated at 37°C and 5% CO_2_ for 18 h and resuspended in GCBL to final OD_600_ of 0.2. Cell suspensions (1.5 mL) were collected and bacteria were lysed by addition of 50 μL of lysis buffer (2% SDS, 4% β-mercaptoethanol, 10% glycerol, 1M Tris-HCl pH 6.8, and 0.01% bromophenol blue) and incubation at 100°C for 10 min. Samples were cooled to room temperature and proteins were digested by addition of 25 μg proteinase K in 10 μL of lysis buffer for 1 h at 60°C. Isolated LOS was resolved on 18% SDS-PAGE gels and visualized by a silver staining procedure [54].

### Adhesion and invasion assays

Human cervical epidermal carcinoma (ME180, ATCC HTB-33) cells were maintained in McCoy’s 5A Medium (Iwakata and Grace Modified; Corning) supplemented with 10% heat inactivated Fetal Bovine Serum (FBS; Gemini Bio-Products). Epithelial cells were seeded in 24 well plates (Greiner BioOne) at 1×10^5^ cells/well and incubated overnight at 37°C in 5% CO_2_. *N*. *gonorrhoeae* strains were grown for 18 h. Bacteria were suspended in McCoy’s 5A Medium supplemented with 10% heat inactivated FBS and containing 0.5 × Kellogg’s supplements. Bacteria were added to the epithelial cells at a multiplicity of infection (MOI) of 10:1 for 1, 2, 3, and 4 h. For adherence assays, cells were washed with PBS and then treated with 1% saponin for 15 min. Lysates were collected and aliquots plated on GCB. Results are expressed as the number of CFUs recovered (*n*=6; mean ±SEMs). For invasion assays, cells were treated with 100 μg/mL of gentamicin for 2 h. After washing the cells extensively, the number of invasive gonococci was quantified by lysing the cells with 1% saponin for 15 min. Aliquots of the lysates were plated on GCB. Results are expressed as the number of CFUs recovered after gentamycin treatment (*n* = 3; mean ±SEMs).

### Competitive murine infections

Female BALB/c mice (6 to 8 weeks old; Charles River Laboratories Inc., Wilmington, MA; NCI Frederick strain of inbred BALB/cAnNCr mice, strain code 555) were treated with water-soluble 17β-estradiol and antibiotics to increase susceptibility to *N*. *gonorrhoeae* [55]. Groups of mice were inoculated vaginally with similar numbers of wild type FA1090 and either isogenic Δ*sliC*, **Δ***sliC*/*P*::*sliC*, or **Δ***sliC*/*P*::sliC* bacteria (total dose, 10^6^ CFU *N*. *gonorrhoeae*; 7 mice/group). Vaginal swabs were collected on days 1, 3, and 5 post-inoculation and suspended in 100 μL GCBL. Vaginal swab suspensions and inocula were cultured quantitatively on GCB agar with streptomycin (total number of CFUs) and GCB with streptomycin and kanamycin (Δ*sliC*, **Δ***sliC*/*P*::*sliC*, or **Δ***sliC*/*P*::sliC* CFU). Results were expressed as the competitive index (CI) using the equation CI = [mutant CFU (output)/wild-type CFU (output)]/[mutant CFU (input)/wild-type CFU (input)]. The limit of detection of 1 CFU was assigned for a strain that was not recovered from an infected mouse. A CI of <1 indicates that the mutant is less fit than the wild type strain. Preliminary experiments were performed in biological duplicates (shown in S3 Fig). Final experiments involving competition assays with three pairs of strains, wild type and Δ*sliC*, wild type and **Δ***sliC/P*::*sliC*, or wild type and **Δ***sliC*/*P*::sliC* were conducted at the same time and on three separate occasions. Statistical analysis was performed using Kruskal-Wallis Dunn’s multiple comparison tests to compare significance of CIs between the **Δ***sliC/*wild type and **Δ***sliC*/*P*::*sliC/*wild type as well as **Δ***sliC*/*P*::sliC*/wild type and **Δ***sliC*/*P*::*sliC/*wild type. Competitive infections were also performed with mixtures of wild-type FA1090 and similar numbers of isogenic **Δ***sliC* in C57BL/6J and B6.129P2-Lyz2tm1(cre)Ifo/J 9 mice, which do not produce lysozyme (Jackson Laboratories). Results from two experiments were combined. Statistical analysis was performed using the Mann-Whitney test to compare statistical significance of CIs between the Δ*sliC*/wild type in C57BL/6J and ΔsliC/wild type in lysozyme knockout mice.

### E-tests

MICs for cefotaxime, azithromycin, tetracycline, polymyxin B, vancomycin, ampicillin, and benzylpenicillin were determined using E-tests (Biomerieux) according to the manufacturer’s recommendations. Each determination was performed on three separate occasions using fresh bacterial cultures and the consensus MICs obtained in at least two trials were reported.

### SDS-PAGE and immunoblotting

Samples of whole cell lysates, protein fractions, intact cells, purified proteins, or vaginal washes were normalized based on either OD_600_, protein concentration, or CFUs as specified in the text. Protein concentration was measured using the DC Protein Assay. SDS-PAGE with loading controls for immunoblotting with differential protein abundances are provided in S4 Fig. Proteins were transferred onto nitrocellulose membranes using a Trans-blot Turbo (Bio-Rad) and detected by immunoblotting as described previously [16]. The immunoblotting analysis was performed using polyclonal rabbit antisera with the following dilutions: anti-SliC (1:20,000), anti-BamA (1:20,000) [16], anti-BamD (1:20,000) [16], anti-SurA (1:10,000) or anti-Zwf (1:10,000) [56] and secondary goat anti-rabit HRP conjugated antibodies (1:10,000) (Bio-Rad).

### Densitometry analysis

SliC abundance during wild type FA1090 infection in female BALB/c mice was determined by densitometry using the volume tool built into Image Lab 5.0 software (Bio-Rad) as described in [42, 57]. Rectangle tool, local background subtraction, and linear regression method were used in the calculations. The amount of SliC on day 1 post-infection was arbitrarily set to 1 and the protein abundance on days 3 and 5 is expressed relative to the SliC level detected on day 1.

### Bioinformatics

Amino acid sequence identity of SliC homologs was assessed by aligning sequences downloaded from NCBI with the Clustal Omega online tool (Clustal 2.1; https://www.ebi.ac.uk/Tools/msa/clustalo/) using the default alignment parameters. A subsequent phylogenetic analysis was performed in Molecular Evolutionary Genetics Analysis (MEGA) version 7.0.26. A maximum likelihood tree was constructed using the Jones-Taylor-Thornton model [58]. Neighbor-Join and BioNJ algorithms were applied to a pairwise-distance matrix derived from the JTT model to obtain an initial tree for the heuristic search. The phylogenies were tested by 500 bootstrap iterations, and the tree with the highest log likelihood is presented.

To analyze single nucleotide polymorphisms of *sliC* (locus NEIP0196), DNA sequences were compared between 44,028 isolates of all *Neisseria spp*. and 4990 isolates of *N*. *gonorrhoeae* deposited into the PubMLST database (http://pubmlst.org/neisseria/ as of October 25, 2017 and April 11, 2018; respectively).

### Statistical analysis

GraphPad Prism’s build-in *t*-test was utilized to determine statistically significant differences between experimental results with the exception of animal studies. A confidence level of 95% was used for all analyses.

### Ethics Statement

Animal experiments were conducted at the Uniformed Services University of the Health Sciences (USUHS) according to the guidelines of the Association for the Assessment and Accreditation of Laboratory Animal Care under a protocol # MIC16-488 that was approved by the University’s Institutional Animal Care and Use Committee. The USUHS animal facilities meet the housing service and surgical standards set forth in the “Guide for the Care and Use of Laboratory Animals” NIH Publication No. 85–23, and the USU Instruction No. 3203, “Use and Care of Laboratory Animals”. Animals are maintained under the supervision of a full-time veterinarian. For all experiments, mice were humanely euthanized by the laboratory technician upon reaching the study endpoint using a compressed CO_2_ gas cylinder in LAM as per the Uniformed Services University (USU) euthanasia guidelines (IACUC policy 13), which follow those established by the 2013 American Veterinary Medical Association Panel on Euthanasia (https://www.usuhs.edu/mps/facilities-resources).

## Supporting information

S1 Table Comparison of SliC from *N*. *gonorrhoeae* FA1090 with other members of MliC/PliC family and *Neisseria* ACP. (PDF)

S2 Table Minimal inhibitory concentration of *N*. *gonorrhoeae* FA1090 wild type, Δ*sliC* measured using E test. (PDF)

S1 Fig Phylogenetic relationships between *Neisseriae* SliC alleles. Maximum likelihood trees, constructed in MEGA7 with the Jones-Taylor-Thornton method, were generated for all SliC alleles (224) identified across Neisseria alleles found in the PubMLST database.
(PDF)

S2 Fig Analysis of single nucleotide polymorphisms of SliC (locus NEIP0196) in *Neisseria* was performed by comparing DNA sequences between 44,0238 *Neisseriae* isolates deposited to the PubMLST (https://pubmlst.org/neisseria/), as of October 25, 2017. (PDF)

S3 Fig Initial competition experiments to assess SliC function of lysozyme inhibition during *N*. *gonorrhoeae* colonization. Groups of mice were inoculated intravaginally with wild-type FA1090 combined with similar CFUs (total dose, 10^6^ CFU *N*. *gonorrhoeae*; 7 mice/group) of isogenic Δ*sliC*. Vaginal swabs were collected on days 1, 3, and 5 post-inoculation and suspended in liquid media. Vaginal swab suspensions and inocula were cultured quantitatively on agar with streptomycin (total number of CFUs) and selective media (supplemented with streptomycin and kanamycin) to enumerate Δ*sliC* CFUs. Results are expressed as the competitive index (CI) using the equation CI = [mutant CFU (output)/wild-type CFU (output)]/[mutant CFU (input)/wild-type CFU (input)]. The limit of detection of 1 CFU was assigned for a strain that was not recovered from an infected mouse. A CI of <1 indicates that the mutant is less fit than the wild type strain. All experiments were performed in biological triplicates and geometric means are shown. Open symbols designate no mutant CFUs recovered.
(PDF)

S4 Fig Loading controls for immunoblotting experiments that showed differential protein abundance. Samples were prepared for SDS-PAGE as described in the text, separated in precast gradient gels and the protein profiles were visualized using colloidal coomassie. Samples in individual experiments matched the corresponding samples used in immunoblotting analyses. (A-B) Loading controls for immunoblotting experiment presented in Fig 1E. (C) Loading controls for immunoblotting analysis shown in Fig 1F. (D) Loading controls for experiment shown in Fig 2C. (E) Loading controls for experiment in Fig 2D. (F) Loading controls for immunoblotting experiment with proteinase K treatment presented in Fig 2E. (G) Loading controls for experiments presented in Fig 6A and 6B. (H) Loading controls for experiments shown in Fig 6C.
(PDF)
