# Supplementary material for: SliC is a surface-displayed lipoprotein that is required for the anti-lysozyme strategy during Neisseria gonorrhoeae infection
Source: PLoS Pathog. 2018 Jul 5;14(7):e1007081. doi: 10.1371/journal.ppat.1007081 (PMC6033465; doi:10.1371/journal.ppat.1007081)
Supplement: S1 Table — (PDF) [file ppat.1007081.s001.pdf]

Supplemental Table S1

Organism with NCBI Accession No.	Amino Acid Identity
Neisseria gonorrhoeae FA1090 SliC [WP_003688168.1]	100%
Neisseria gonorrhoeae ACP [WP_010951375.1]	14.16%
Neisseria meningitidis ACP [NP_275083.1]	14.41%
Neisseria weaveri SliC [WP_004282409.1]	45.3%
Neisseria lactamica SliC [WP_003713844.1]	97.62%
Neisseria meningitids SliC [WP_002219055.1]	96.03%
Salmonella enterica Typhimurium PliC [Q8ZPY8]	16.98%
Serratia marcescens MliC [ALE95990.1]	15.46%
Edwardsiella tarda MliC [ADM41632.1]	27.27%
Klebsiella pneumoniae MliC [CDQ54222.1]	19.42%
Salmonella enterica Typhimurium MliC [NP_460410.1]	19.42%
Shigella flexneri MliC [CDX07019.1]	21.05%
Escherichia coli MliC [AJE56069.1]	18.81%
Avian Pathogenic *E. coli* MliC [AKK39057.1]	20.39%
Pseudomonas aeruginosa MliC [AAG04256.1]	23.33%
