# Supplementary material for: SliC is a surface-displayed lipoprotein that is required for the anti-lysozyme strategy during Neisseria gonorrhoeae infection
Source: PLoS Pathog. 2018 Jul 5;14(7):e1007081. doi: 10.1371/journal.ppat.1007081 (PMC6033465; doi:10.1371/journal.ppat.1007081)
Supplement: S2 Table — (PDF) [file ppat.1007081.s002.pdf]

Supplemental Table S2

**Antibiotic**	**wild type**	**Δ*sliC***
Polymyxin B	128	128
Azithromycin	0.064	0.064
Ceftriaxone	0.004	0.004
Ampicillin	0.125	0.125
Tetracycline	0.125	0.125
Penicillin	0.064	0.064
