# Supplementary figures and images for: SliC is a surface-displayed lipoprotein that is required for the anti-lysozyme strategy during Neisseria gonorrhoeae infection
Source: PLoS Pathog. 2018 Jul 5;14(7):e1007081. doi: 10.1371/journal.ppat.1007081 (PMC6033465; doi:10.1371/journal.ppat.1007081)

Supplemental Figure S1

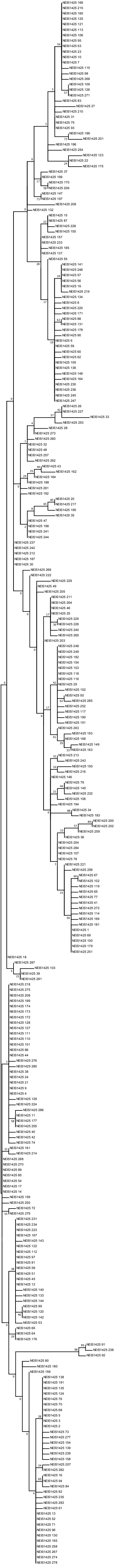

Supplement: S1 Fig — Maximum likelihood trees, constructed in MEGA7 with the Jones-Taylor-Thornton method, were generated for all SliC alleles (224) identified across Neisseria alleles found in the PubMLST database. (PDF) [file ppat.1007081.s003.pdf]

### Supplemental Figure S3

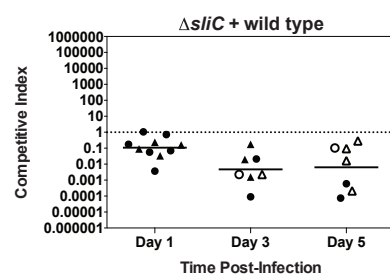

Supplement: S3 Fig — Groups of mice were inoculated intravaginally with wild-type FA1090 combined with similar CFUs (total dose, 106 CFU N. gonorrhoeae; 7 mice/group) of isogenic ΔsliC. Vaginal swabs were collected on days 1, 3, and 5 post-inoculation and suspended in liquid media. Vaginal swab suspensions and inocula were cultured quantitatively on agar with streptomycin (total number of CFUs) and selective media (supplemented with streptomycin and kanamycin) to enumerate ΔsliC CFUs. Results are expressed as the competitive index (CI) using the equation CI = [mutant CFU (output)/wild-type CFU (output)]/[mutant CFU (input)/wild-type CFU (input)]. The limit of detection of 1 CFU was assigned for a strain that was not recovered from an infected mouse. A CI of <1 indicates that the mutant is less fit than the wild type strain. All experiments were performed in biological triplicates and geometric means are shown. Open symbols designate no mutant CFUs recovered. (PDF) [file ppat.1007081.s005.pdf]

# Supplemental Figure S4

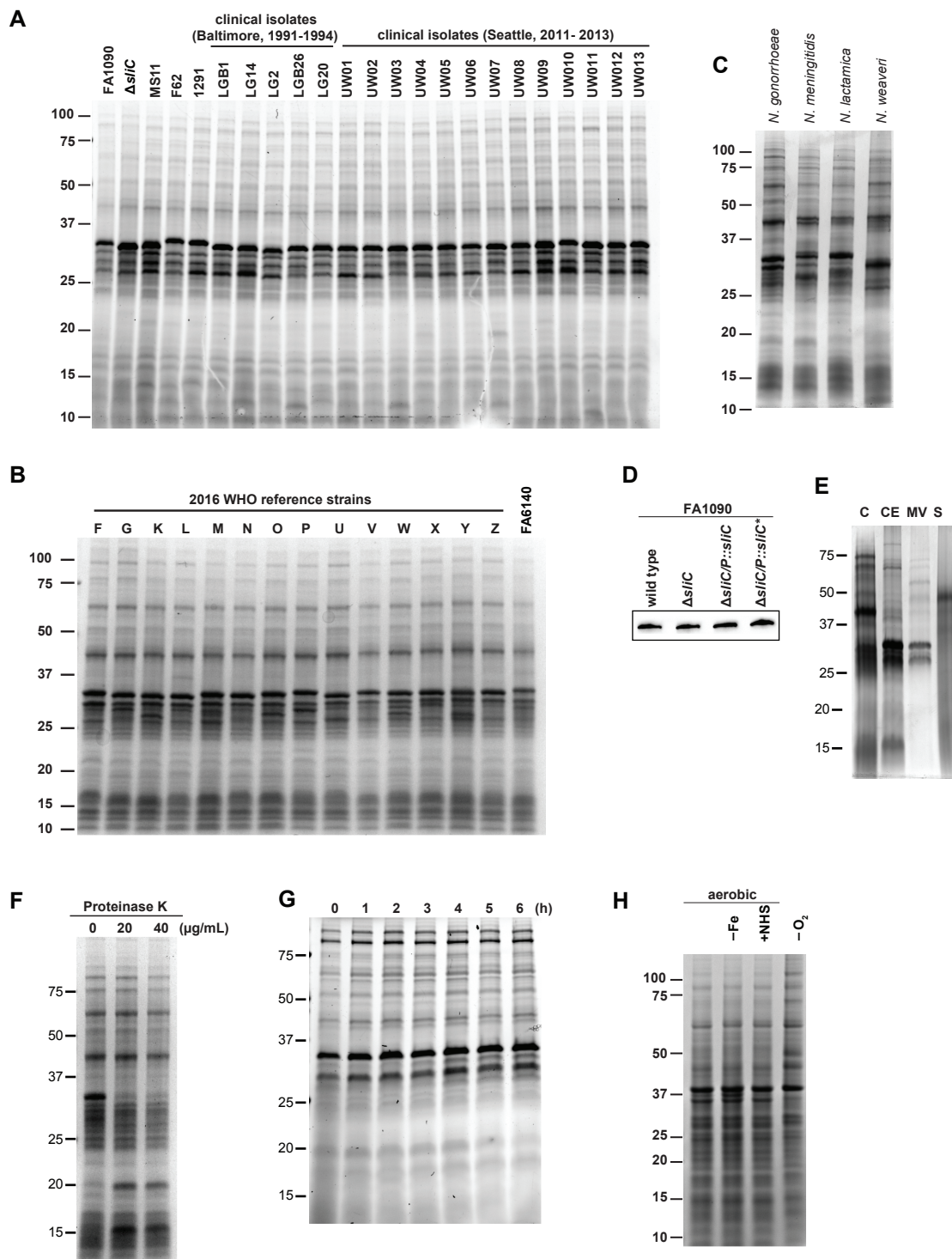

Supplement: S4 Fig — Samples were prepared for SDS-PAGE as described in the text, separated in precast gradient gels and the protein profiles were visualized using colloidal coomassie. Samples in individual experiments matched the corresponding samples used in immunoblotting analyses. (A-B) Loading controls for immunoblotting experiment presented in Fig 1E. (C) Loading controls for immunoblotting analysis shown in Fig 1F. (D) Loading controls for experiment shown in Fig 2C. (E) Loading controls for experiment in Fig 2D. (F) Loading controls for immunoblotting experiment with proteinase K treatment presented in Fig 2E. (G) Loading controls for experiments presented in Fig 6A and 6B. (H) Loading controls for experiments shown in Fig 6C. (PDF) [file ppat.1007081.s006.pdf]
