# Supplementary material for: SliC is a surface-displayed lipoprotein that is required for the anti-lysozyme strategy during Neisseria gonorrhoeae infection
Source: PLoS Pathog. 2018 Jul 5;14(7):e1007081. doi: 10.1371/journal.ppat.1007081 (PMC6033465; doi:10.1371/journal.ppat.1007081)
Supplement: S2 Fig — (PDF) [file ppat.1007081.s004.pdf]

Supplemental Figure S2

The color codes represent the percentage of alleles that have a particular nucleotide at each position.

224 alleles included in analysis. 157 polymorphic sites found.

Key: 0 - 10 | >10 - 20 | >20 - 30 | >30 - 40 | >40 - 50 | >50 - 60 | >60 - 70 | >70 - 80 | >80 - 90 | >90 - 100

```
1        10        20        30        40        50        60        70        80        90        100
ATGAATATCAGGTTTTTCGCGCTGACCGTACCGGTTTTGTCTTTGGCGGCCTGTGCCGTGCCGGAGGCGTATGATGGCGGCGGACGCGGGCATATGCCGC
   TG  C   A     TTTA   G T  GTTC        CC A T   TG  A TACA  A  AATAG      AA  AA  GAA   T     TTAT
                          A     A                                            T   C   TT
101      110       120       130       140       150       160       170       180       190       200
CCGTTCAAAACCAAGCCGGCCCGGACGATTTTCGGGCGTTTTCCTGCGAGAACGGTTTGTCTGTGCGCGTCCGCCATTTGGACGGCGGCAAAATCGCGTT
 TA C  C  T   ATTCATATAACA G GG  AAATA     AA TA       G        A AGA T  TAGG  A  TAAT  A GGG T TA
 A                  T T  T          A                              T                    T       A
                    G
201      210       220       230       240       250       260       270       280       290       300
GCGGCTGGACGGCAGGCGTGCCGTCCTCTCTTCCGACGTTGCCGCATCCGGCGAACGCTATACCGCCGAACACGGTTTGTTCGGAAACGGAACCGAGTGG
A ATT A  TAAT AAT C  TA TT T     T C      T     T  A    AT   G T  A  GA T   C A  T    G  CG T
         G      G G        G                       T         C
301      310       320       330       340       350       360       370       380
CACCAGAAAGGCGGCGAAGCCTTTTTCGGCTTTACCGATGCCTACGGCAATTCGGTCGAAACTTCCTGCCGCGCCCGTTAA
  T        AA T      G C  T  T     A         T     CTAA T     C T   TT TATT  C G
           T                                                            T
```
